# Developing Allosteric Inhibitors of SARS‐CoV‐2 RNA‐Dependent RNA Polymerase

**DOI:** 10.1002/cmdc.202400367

**Published:** 2024-10-03

**Authors:** Artem Chayka, Matěj Danda, Alžběta Dostálková, Vojtěch Spiwok, Anna Klimešová, Marina Kapisheva, Michala Zgarbová, Jan Weber, Tomáš Ruml, Michaela Rumlová, Zlatko Janeba

**Affiliations:** ^1^ Institute of Organic Chemistry and Biochemistry of the Czech Academy of Sciences Flemingovo nám. 2 16000 Prague 6 Czech Republic; ^2^ Department of Biotechnology University of Chemistry and Technology Prague, Technická 5 16628 Prague 6 Czech Republic; ^3^ Department of Biochemistry and Microbiology University of Chemistry and Technology Prague, Technická 5 16628 Prague 6 Czech Republic; ^4^ Department of Genetics and Microbiology Charles University, Faculty of Sciences Viničná 5 12844 Prague 2 Czech Republic

**Keywords:** allosteric inhibitor, SARS-CoV-2, RdRp; remdesivir, SAR study, scaffold hopping

## Abstract

The use of Fpocket and virtual screening techniques enabled us to identify potential allosteric druggable pockets within the SARS‐CoV‐2 RNA‐dependent RNA polymerase (RdRp). Of the compounds screened, compound **1** was identified as a promising inhibitor, lowering a SARS‐CoV‐2 RdRp activity to 57 % in an enzymatic assay at 10 μM concentration. The structure of compound **1** was subsequently optimized in order to preserve or enhance inhibitory activity. This involved the substitution of problematic ester and aromatic nitro groups with more inert functionalities. The *N*,*N’*‐diphenylurea scaffold with two NH groups was identified as essential for the compound's activity but also exhibited high toxicity in Calu‐3 cells. To address this issue, a scaffold hopping approach was employed to replace the urea core with potentially less toxic urea isosteres. This approach yielded several structural analogues with notable activity, specifically 2,2’‐bisimidazol (in compound **55** with residual activity RA=42 %) and (1*H*‐imidazol‐2‐yl)urea (in compounds **59** and **60**, with RA=50 and 28 %, respectively). Despite these advances, toxicity remained a major concern. These compounds represent a promising starting point for further structure‐activity relationship studies of allosteric inhibitors of SARS‐CoV‐2 RdRp, with the goal of reducing their cytotoxicity and improving aqueous solubility.

## Graphical abstract

Fpocket enables the identification of potential allosteric druggable pocket within the SARS‐CoV‐2 RNA‐dependent RNA polymerase (RdRp), virtual screening leads to an identification of compound **1** as a promising inhibitor of a SARS‐CoV‐2 RdRp activity with residual activity RA=57 % in an enzymatic assay (at 10 μM concentration) and a subsequent SAR study offers compound **60** with RA=28 %.

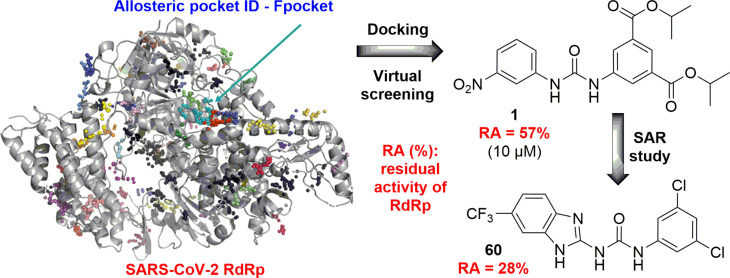

## Introduction

In late 2019, severe acute respiratory syndrome coronavirus 2 (SARS‐CoV‐2) initiated the global pandemic of COVID‐19. Although several drugs have been approved for its treatment (at first as emergency use authorization) and reduced pathogenicity has been observed with newer variants of the virus, COVID‐19 remains a major threat to health and the healthcare systems around the world.^[1]^

One of the main strategies used to develop antiviral drugs is to inhibit essential enzymes in the virus lifecycle.^[2]^ RNA‐dependent RNA polymerase (RdRp) of SARS‐CoV‐2 (non‐structural protein 12 or nsp12) has been considered an ideal target for inhibition because of its crucial role in the replication of the virus and its absence in humans.[^3^, ^4^] In general, there are two main types of inhibitors of RdRp: catalytic site and allosteric site inhibitors.[^5^, ^6^]

The catalytic site of RdRp is relatively conserved among all RNA viruses including SARS‐CoV‐2.^[7]^ This conservation is a good basis for the repurposing of broad‐spectrum RdRp inhibitors, such as favipiravir, remdesivir, and molnupiravir.[^8^, ^9^] An open‐label randomized trial in China showed that while favipiravir is able to relieve fever in COVID‐19 patients, it did not significantly improve the clinical recovery rate.^[10]^ The Adaptive COVID Treatment Trial (ACTT) of remdesivir demonstrated a small reduction in mortality among hospitalized adults from 15.2 % to 11.4 % compared with placebo,^[11]^ while the Hubei (China) study reported no statistically significant improvement compared with placebo.^[12]^ In addition, after conducting its own clinical trials and reviewing three other clinical trials, involving more than 7,000 people in total, the WHO at first did not recommend the use of remdesivir in COVID‐19 patients.^[13]^ Molnupiravir has been better received, due to the reduction of post‐hospitalization mortality rate from 9.7 % to 6.8 % compared to placebo in a 30‐day‐long study in patients with moderate COVID‐19.^[14]^ Unfortunately, the effectiveness of catalytic site inhibitors is highly diminished because of the emergence of drug‐resistant variants of the virus due to the activity of the viral proofreading exonuclease nsp10/14 complex.^[15]^ This problem can be potentially alleviated by co‐administration of the active site inhibitor with an allosteric inhibitor.

So far, no allosteric inhibitors of SARS‐CoV‐2 RdRp have been reported. There are few computational studies exploring allosteric sites of RdRp^[16]^ and one study suggesting flavonoid‐based allosteric inhibitors.^[17]^

Based on this knowledge and information about the RdRp protein structure, we have identified allosteric pockets with the highest probability of affecting the main catalytic pocket. We performed a virtual screening of commercially available compounds, and after refinement, we purchased a series of compounds from which we identified one active compound as a starting point for the search of potential allosteric SARS‐CoV‐2 RdRp inhibitors.

## Results and Discussion

### Structural Scaffold Identification

**Figure 1:**
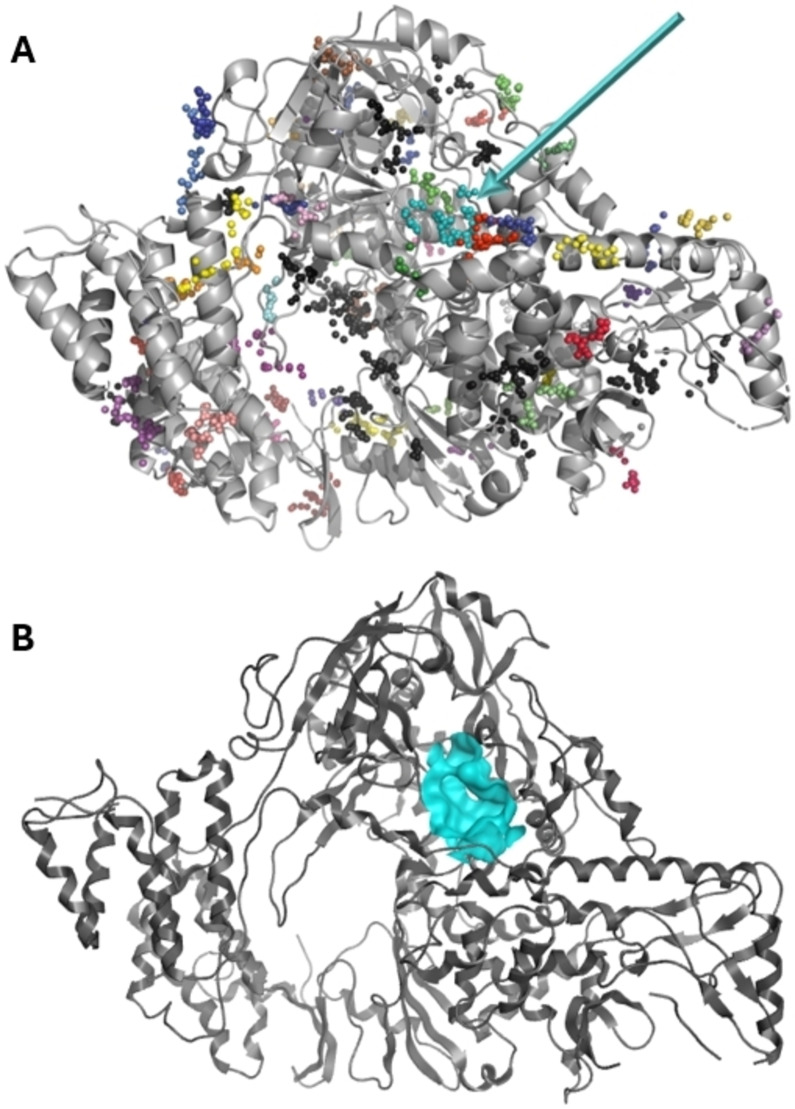
Identification of potential druggable pockets on SARS‐CoV‐2 RdRp protein CryoEM structure (PDB ID: 6 M71) using Fpocket. A: Pocket 1 is labelled with the blue arrow. Other pockets are depicted as series of spheres of the same color. B: Surface of Pocket 1 is highlighted in blue.

The identification of a novel binding pocket in the target protein is a challenging task, especially for binding sites that allosterically influence the orthosteric site. To identify potential druggable pockets and to predict their druggability, we scanned the surface of SARS‐CoV‐2 RdRp protein CryoEM structure (PDB ID: 6 M71)^[18]^ using Fpocket. The Fpocket tool identified 73 pockets with a druggability score ranging from 0.000 to 0.842 (0‐least druggable, 0.842‐most druggable, Figure 1).

The selected pocket, further referred to as Pocket 1 (Figure 1), was ranked as number 4 in the druggability score (0.644). It was selected for further studies as a compromise of its size, druggability, and active site proximity. The residues forming Pocket 1 are Asn‐314, Val‐315, Ser‐318, Thr‐319, Phe‐326, Gly‐327, Tyr‐346, His‐347, Phe‐348, Arg‐349, Glu‐350, Asn‐459, Leu‐460, Pro‐461, Thr‐462, Asn‐628, Ser‐664, Val‐675, Lys‐676, Pro‐677.

**Figure 2:**
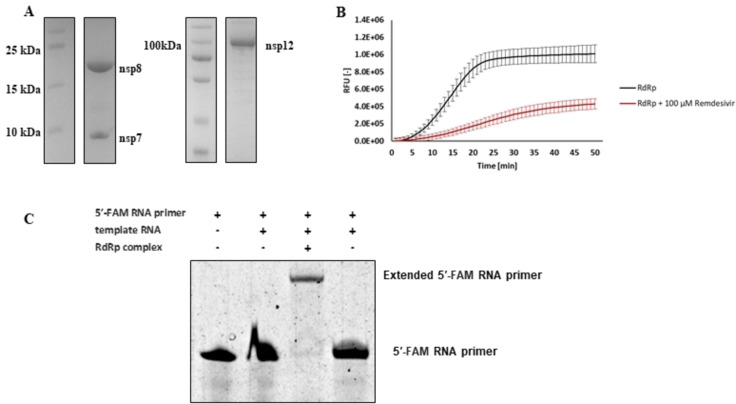
Enzymatic assay for the SARS‐CoV‐2 RdRp activity assessment (A) SDS‐PAGE analysis of purified recombinant non‐structural proteins (nsp7, nsp8, and nsp12) comprising the SARS‐CoV‐2 RdRp; the left lanes show the molecular weight markers. (B) RdRp polymerase activity determined by the fluorometric RNA extension assay. The black line represents a non‐inhibited reaction. In the presence of a competitive RdRp inhibitor remdesivir (red line) an inhibitory effect was observed. (C) TBE‐UREA‐PAGE verification of a dsRNA product replicated from the FAM labelled RNA primer under the same conditions as in the fluorometric assay.

SARS‐CoV‐2 RdRp (nsp12) requires two additional co‐factor subunits, non‐structural proteins 7 (nsp7) and 8 (nsp8), which significantly enhance its polymerase activity, and form the minimal RdRp complex.^[19]^ For *in vitro* studies of the RdRp activity, we purified all the proteins of the minimal RdRp complex, i. e., nsp12 (RdRp), nsp7, and nsp8 (Figure 2A). To assess the polymerase activity of the SARS‐CoV‐2 RdRp complex, we modified a published fluorometric RNA extension assay to effectively screen tens of compounds for their ability to inhibit the synthesis of double‐stranded RNA (dsRNA).^[20]^ Our polymerase assay is based on the detection of fluorescence emitted by SYBR Green I which interacts exclusively with double‐stranded nucleic acid ensuring the detection of the desired products of RNA replication. We also verified the applicability of the assay for inhibitor screening by testing the reaction in the presence of 100 μM GS‐441524‐triphosphate, the active metabolite derived from antiviral drug remdesivir (Figure 2B). Notably, GS‐441524 is an adenosine nucleoside analogue that can be incorporated into the nascent RNA strand. In the designed enzymatic assay, GS‐441524‐triphosphate can steadily compete with ATP for the NTP‐binding site. To achieve an inhibitory effect comparable to that of possible allosteric inhibitors, a higher concentration of GS‐441524‐triphosphate is required.^[21]^

Furthermore, the continuous monitoring of fluorescence over time (Figure 2B) allowed us to monitor the kinetics of RNA replication compared to the gel‐based assay where only the final product is detected after an incubation.^[22]^ The gel‐based assay was, however, utilized to verify products synthesized during the polymerase reaction (Figure 2C). Tested compounds were sorted based on the residual activity (RA, in %) of the SARS‐CoV‐2 RdRp complex, which was calculated as the area under the amplification curve (AUC) of the reaction in the presence of the compound divided by the AUC of the non‐inhibited reaction.

**Figure 3:**
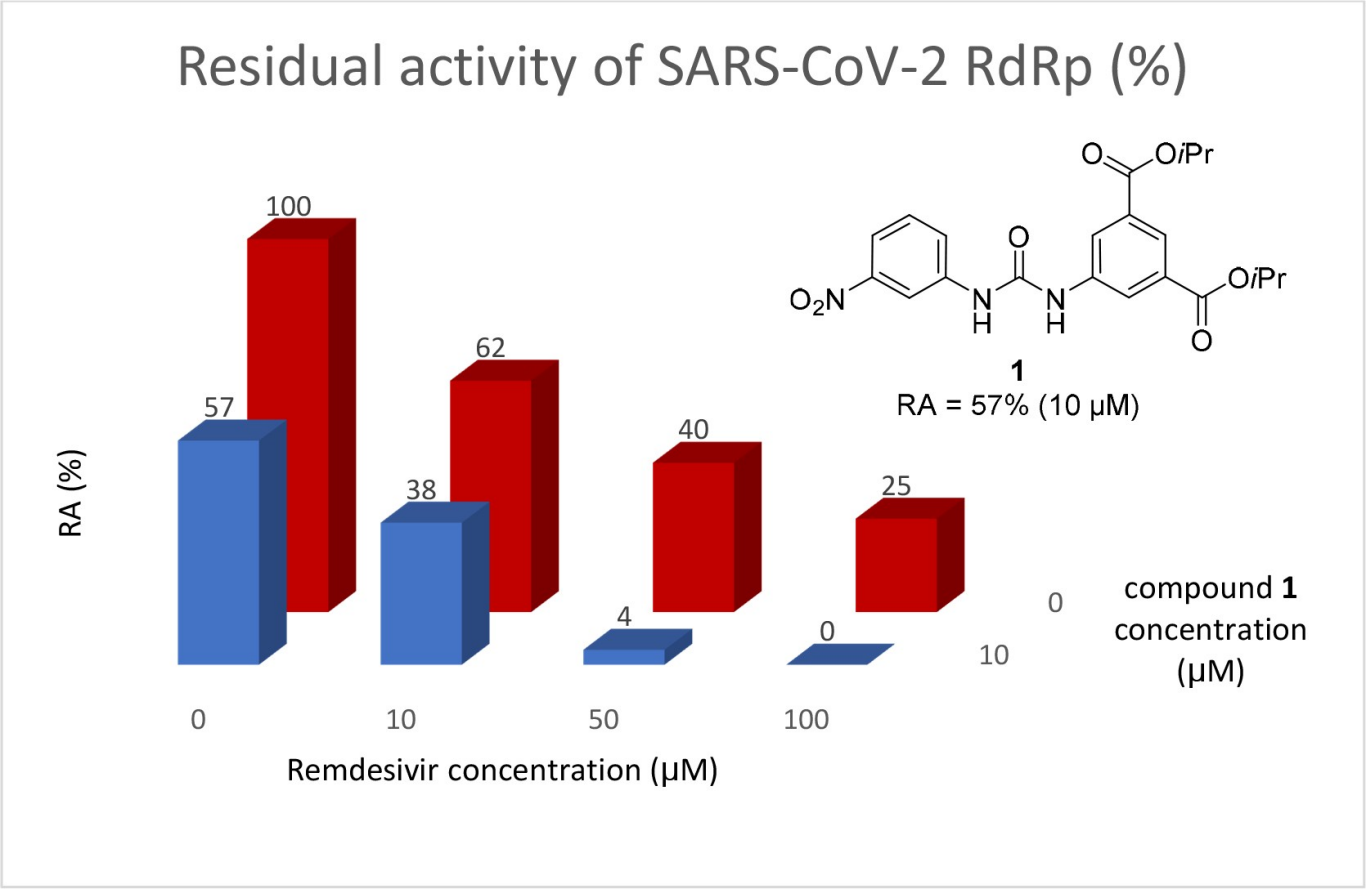
Structure of compound **1** and its effect at 10 μM concentration on residual activity (RA) of SARS‐CoV‐2 RdRp in combination with various concentrations of remdesivir (in blue). The inhibitory effect of remdesivir alone is shown in red.

On Pocket 1 (Figure 1), a virtual screening of 68,380 commercially available compounds was performed and 20 compounds were selected based on the docking score, originality, and visual inspection. These compounds (labelled as compound **1** and compounds **A**–**S**, Table S1) were purchased and tested using the fluorescence‐based RdRp enzymatic assay. Of the compounds tested, compound **1** (Figure 3) reduced the activity of the enzyme (residual activity, RA) to 57 % of the initial value in a 10 μM screening. Moreover, the compound potentiated the effect of active site inhibitor remdesivir, which further supported possible allosteric modulation by compound **1**.

The inhibitory activity of compound **1** in SARS‐CoV‐2 infected Calu‐3 cells could not be determined because of its apparent high toxicity (CC_50_=23 μM) ranging in a similar concentration as the effective inhibitory concentration (Table 1). Moreover, the compound was poorly soluble in water. Thus, an extensive optimization of the compound properties was required.

**Table 1:** Anti‐SARS‐CoV‐2 activity of **1**, endovion, and remdesivir on Calu‐3 cells.

Compound	EC_50_ [μM]	CC_50_ [μM]
**1**	>23	23
Endovion	>64	>64
Remdesivir	0.11	>3.2

### Initial Modifications of the Scaffold

**Figure 4:**
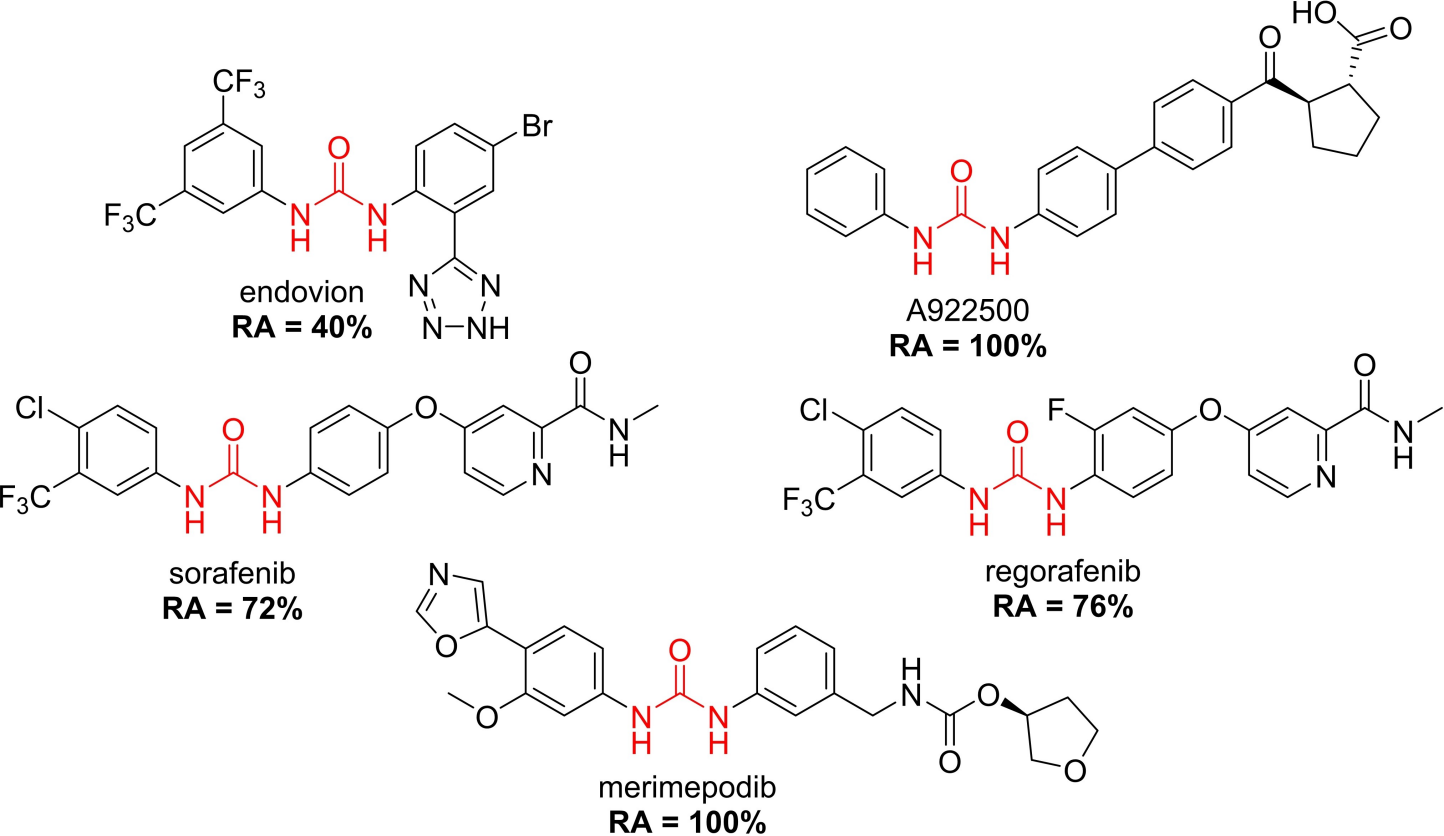
*N*,*N*’‐diphenylurea derivatives reported to inhibit (with or without remdesivir) SARS‐CoV‐2 in cells and determination of their residual activity (RA) of SARS‐CoV‐2 RdRp.

Several compounds with a similar structure to compound **1** (our selected hit) have been reported to inhibit SARS‐CoV‐2 replication in cells (Figure 4). Merimepodib in combination with remdesivir was able to completely suppress viral replication.[^23^, ^24^] Unfortunately, this combination tested in clinical trials against COVID‐19 exhibited serious safety issues.^[25]^ Sorafenib strongly inhibited cell infection^[26]^ and was indirectly confirmed to positively affect cancer patients with COVID‐.^[27]^ Several other compounds, such as regorafenib, A922500, and endovion have been reported to be active in cell assays against the virus.[^26^, ^28^, ^29^] All of these compounds share the same structural motif, the *N*,*N*’‐diphenylurea core (Figure 4), and probably share the same mechanism of action. However, all of them, except for merimepodib, belong to the class of cancerostatics and are, thus, highly toxic.

All above mentioned compounds (Figure 4) were purchased and evaluated in our RdRp assay. We found, that they were either inactive (RA=100 %) or less potent (RA>70 %) compared to our compound **1** with RA=57 %. The exception was endovion (RA=40 %), which exhibited better *in vitro* inhibitory potency compared to compound **1**, but compound **1** appeared to be more potent in the antiviral assay on Calu‐3 cells (Table 1). Unfortunately, the inhibitory activity of both compounds was connected with cytotoxicity.

Both endovion and compound **1** were poorly soluble in water, which is an important aspect to deal with in designing drug‐like molecules. We decided to develop a novel SARS‐CoV‐2 RdRp inhibitor based on structures of both compound **1** and endovion, as the starting point.

### Synthesis of the First Series of Derivatives

The first goal was to identify the pharmacophore of the compounds and to remove/replace potentially problematic groups from compound **1**: either the aromatic nitro group (potentially toxic in biological systems) or the isopropyl ester groups (labile and overly bulky) or both.

**Scheme 1:**
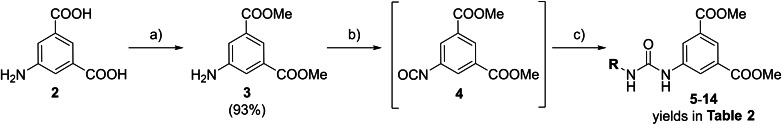
Synthesis of compounds **5**–**14**. Reagents and conditions: a) SOCl_2_, MeOH, 60 °C; b) triphosgene, Et_3_N, DCM, 0 °C‐RT; c) R‐NH_2_ (for R see Table 2), DCM, 60 °C.S.

Derivatives of compound **1** with the replaced 3‐nitrophenyl group (and with retained isophthalate moiety in the methyl ester form) were synthesized from 5‐aminoisophthalic acid (**2**), which was converted into dimethyl ester **3** and afterwards into dimethyl 5‐isocyanoisophthalate (**4**). Compound **4** (without isolation) reacted with various anilines to afford desired compounds **5**–**14** (Scheme 1, Table 2).

**Table 2:** Yields of compounds **5**–**14** (Scheme 1).^[a]^

Compd	R	Yield (%)	Comp	R	Yield (%)
**5**	Bu‐	55	**10** 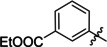		53
**6**	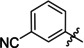	33	**11** 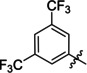		67
**7**	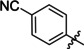	22	**12** 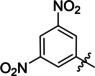		39
**8**	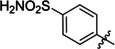	35	**13** 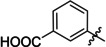		54
**9**	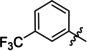	59	**14** 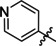		60

[a] Isolated yields.

**Scheme 2:**
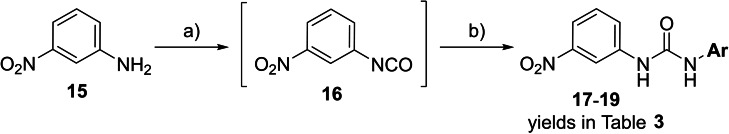
Synthesis of compounds **17**–**19**. Reagents and conditions: a) triphosgene, Et_3_N, DCM, 0 °C‐RT; b) Ar‐NH_2_ (for Ar see Table 3), DCM, 60 °C.

Compounds with retained 3‐nitrophenyl moiety were synthesized from 3‐nitroaniline (**15**) by its conversion into 1‐isocyanato‐3‐nitrobenzene (**16**), which was without isolation reacted with various anilines to afford compounds **17**–**19** (Scheme 2, Table 3).

**Table 3:** Yields of compounds **17**–**19** (Scheme 2).^[a]^

Compd	Ar	Yield (%)	Comp	Ar	Yield (%)
**17**	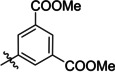	10	**19** 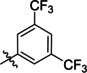		61
**18**	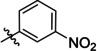	27			

[a] Isolated yields.

**Scheme 3:**
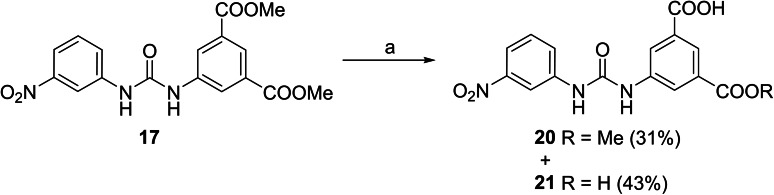
Synthesis of compounds **20** and **21**. Reagents and conditions: a) LiOH, H_2_O/dioxane, RT.

Corresponding monoester **20** and isophthalic acid derivative **21** were prepared by basic hydrolysis of compound **17** (Scheme 3).

Next, commercially available compounds **22**–**44** (structures are shown in Tables 4, 5, 6) were purchased from Vitas‐M Laboratory (Champaign, IL, USA).

**Table 4:** Activity of SARS‐CoV‐2 RdRp complex in the presence of compounds **5**–**14** and **22**–**28** (bearing the dialkyl isophthalate ring) shown as residual activity (RA) in %.^[a]^

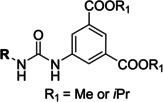							
Compd	R	R1	RA (%)	Comp	R	R1	RA (%)
**5**	Bu‐	Me	100	**14**	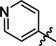	Me	100
**6**	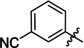	Me	100	**22**	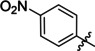	Me	50
**7**	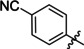	Me	100	**23**	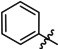	Me	100
**8**	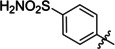	Me	100	**24**	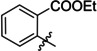	*i*Pr	100
**9**	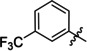	Me	70	**25**	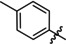	Me	100
**10**	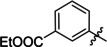	Me	100	**26**	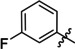	Me	100
**11**	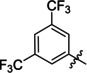	Me	65	**27**	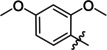	*i*Pr	100
**12**	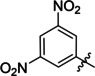	Me	100	**28**	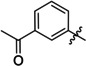	*i*Pr	100
**13**	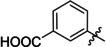	Me	100				

[a] RA (%) is the residual activity of the SARS‐CoV‐2 RdRp complex.

**Table 5:** Activity of SARS‐CoV‐2 RdRp complex in the presence of compounds **17**–**21** and **29**–**40** (bearing 3‐nitrophenyl ring) shown as residual activity (RA) in %.^[a]^

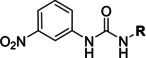					
Compd	Substituent	RA (%)	Comp	Substituent	RA (%)
**17**	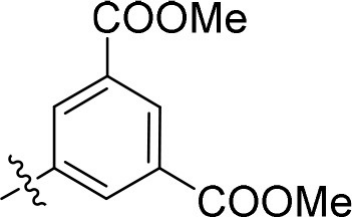	51	**33**	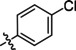	100
**18**	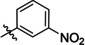	100	**34**	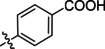	100
**19**	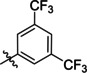	62	**35**	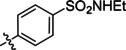	100
**20**	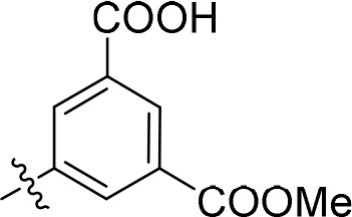	100	**36**	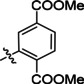	100
**21**	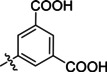	100	**37**	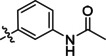	100
**29**	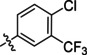	58	**38**	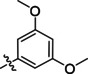	100
**30**	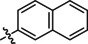	73	**39**	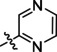	100
**31**	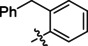	78	**40**	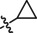	100
**32**	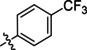	100			

[a] RA (%) is the residual activity of the SARS‐CoV‐2 RdRp complex.

**Table 6:** Activity of SARS‐CoV‐2 RdRp complex in the presence of compounds **41**–**44** shown as residual activity (RA) in %.^[a]^

Compd	Structure	RA (%)
**41**	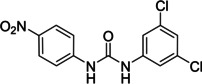	40
**42**	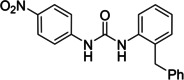	63
**43**	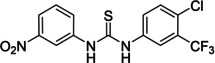	77
**44**	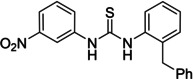	100
**47**	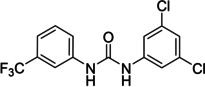	45

[a] RA (%)s is the residual activity of the SARS‐CoV‐2 RdRp complex.

### Biological Activity of the First Series of Compounds

The first series of compounds was divided into three groups: compounds with a dialkyl isophthalate ring preserved (Table 4), compounds with preserved 3‐nitrophenyl ring (Table 5), and compounds with multiple structural modifications compared to the parent compound **1** (Table 6).

The replacement of the 3‐nitrophenyl ring was challenging and only rings with similar electronic effects (i. e., bearing electron‐withdrawing group, EWG), namely 3‐trifluoromethylphenyl, 3,5–di(trifluoromethyl)phenyl, and 4‐nitrophenyl, afforded active compounds with RA=50–70 % (derivatives **9**, **11**, and **22**, respectively, Table 4). On the other hand, dialkyl isophtalate (Table 5) could be replaced by rings such as 3,5‐di(trifluoromethyl)phenyl (in compound **19**), 4‐chloro‐3‐(trifluoromethyl)phenyl (in **29**), 2‐naphtyl (in **30**), and 2‐benzylphenyl (in **31**), to preserve activity (RA=58–78 %). Similarly to derivative **22** (Table 4), compounds **41** and **42** (Table 6), bearing the 4‐nitrophenyl moiety exhibited good activity, where compound **41** (RA=40 %) was the most potent derivative so far. Interestingly, the removal of one or both methyl ester groups from active compound **17** (RA=51 %) led to a complete loss of activity in compounds **20** and **21**, suggesting that polar groups on the phenyl ring are not tolerated. Surprisingly, also thiourea‐based compound **43** (Table 6) decreased the activity of the SARS‐CoV‐2 RdRp complex with RA=77 %.

**Scheme 4:**
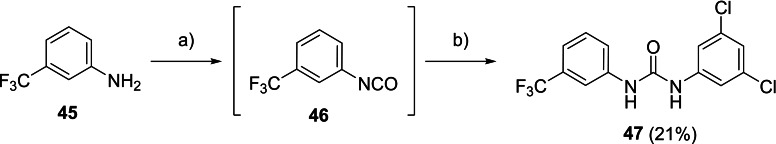
Synthesis of compound **47**. Reagents and conditions: a) triphosgene, Et_3_N, DCM, 0 °C–RT; b) 3,5‐dichlorophenyl, DCM, 60 °C.

The above data suggested that the 3‐trifluoromethylphenyl and 3,5‐dichlorophenyl moieties represented suitable replacements for the 3‐nitrophenyl and dialkyl isophthalate moieties (both present in compound **1**), respectively. Thus, compound **47** (Scheme 4) was designed to combine both these moieties while eliminating the potentially problematic nitro function and the ester groups. Compound **47** was prepared in a 21 % yield using the above‐developed procedure starting from 3‐(trifluoromethyl)aniline (**45**) *via* isocyanato derivative **46**. Compound **47** proved to be a potent inhibitor of the SARS‐CoV‐2 RdRp complex (RA=45 %, Table 6) and a promising new lead for further research.

Altogether, several important structure‐activity relationship (SAR) features were observed. From the comparison of compounds **1** (RA=57 %) vs. **22** (RA=50 %) and **31** (RA=78 %) vs. **42** (RA=63 %) could be concluded that a strong EWG (in this case nitro group) in *para* instead of *meta* position of the phenyl ring improved the activity of the compounds. Also, the comparison of compounds **29** (RA=58 %) vs. **43** (RA=77 %) and **31** (RA=78 %) vs. **44** (RA=100 %) suggested urea to be a more favourable structural moiety compared to thiourea. Thus, it could be concluded that the acidity of NH groups of the central linker (urea or thiourea) is proportional to the activity of the compounds.

**Scheme 5:**
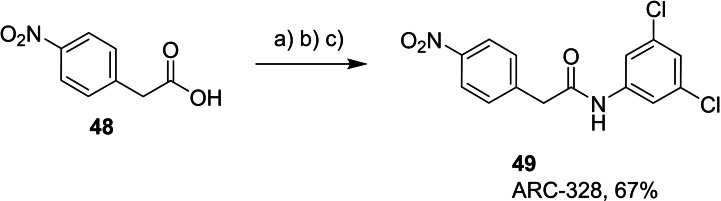
Synthesis of compound **49**. Reagents and conditions: a) SOCl_2_ neat, RT; b) solvent removal; c) 3,5‐dichloroaniline, Et_3_N, DCM, RT.

**Scheme 6:**
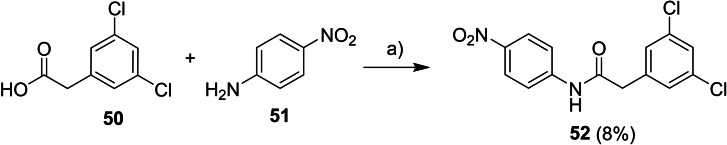
Synthesis of compound **52**. Reagents and conditions: a) HBTU, Et_3_N, DMF, RT.

To find out if both central NH groups are essential in urea‐based compounds, the most potent compound of the 1^st^ series, derivative **41**, was further modified by a replacement of each NH group for methylene. Compounds **49** and **52** were synthesized, where the NH group was removed from one or the other side of the urea (Schemes 5 and 6).

**Figure 5:**
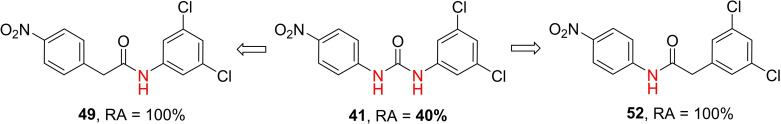
Comparison of activity of SARS‐CoV‐2 RdRp complex (as residual activity (RA) in %) for compounds **41**, **49**, and **52**. Both NH groups (in red) are essential for the activity.

The presence of both NH groups was proved to be crucial since the removal of each of them from compound **41** led to a complete loss of activity on the SARS‐CoV‐2 RdRp complex (Figure 5).

Selected compounds from the 1^st^ series (namely **1**, **11**, **19**, **24**, and **47**) were tested against SARS‐CoV‐2 in Calu‐3 cells (Table 7). It appeared, that the aromatic nitro group was not the main source of toxicity as **24** and **1** exhibited a similar level of toxicity. Reduction of toxicity of these compounds appears to be a major challenge that needs to be addressed.

**Table 7:** Activity of selected compounds (the first series) in Calu‐3 cells infected with SARS‐CoV‐2.

Compd	EC_50_ [μM]	CC_50_ [μM]
**1**	>23	23
**9**	>10	10.6
**11**	>5.0	5.1
**19**	>3.0	3.4
**47**	>0.49	0.49
Remdesivir	0.11	>5.0

In summary, the key structural feature of studied compounds consists of *N*,*N*’‐diphenylurea, where both aryl moieties carry potent EWGs, e. g. 3‐nitrophenyl, 4‐nitro‐phenyl, 3,5‐dichlorophenyl or 3‐(trifluoromethyl)phenyl. Elimination of one of the NH groups in compound **41** (RA=40 %) resulted in a complete loss of inhibitory activity (compounds **49** and **52** with RA=100 %). Furthermore, urea derivatives exhibited higher activity than thiourea (compound **29** with RA=58 % vs. compound **43** with RA=77 %, respectively), and *para*‐EWGs on aromatic rings proved more beneficial for the potency compared to *meta*‐EWGs, which suggested that the inhibitory activity of the compounds was directly proportional to the acidity of the two NH groups. Unfortunately, the prepared compounds (including **47**) exhibited substantial toxicity to Calu‐3 cells.

### Design and Synthesis of the Second Series of Derivatives–Urea Isosteres

The toxicity of enzyme inhibitors can be reduced by the scaffold hopping approach.[^30^, ^31^] This is a computational approach in which some of the desired properties of a scaffold are preserved while its core structure is altered.^[32]^ Typical techniques involve shape matching and pharmacophore searching. Based on various criteria, these techniques compare the current core structure with a library of fragments and, in this way, identify the structure that best mimics the parent core.^[32]^ To date, however, scaffold hopping has not been applied as a strategy for reducing the toxicity of allosteric inhibitors of SARS‐CoV‐2 RdRp complex. In order to design novel compounds, we applied pharmacophore search to identify new analogues of the *N*,*N*’‐biphenyl urea scaffold.

**Figure 6:**
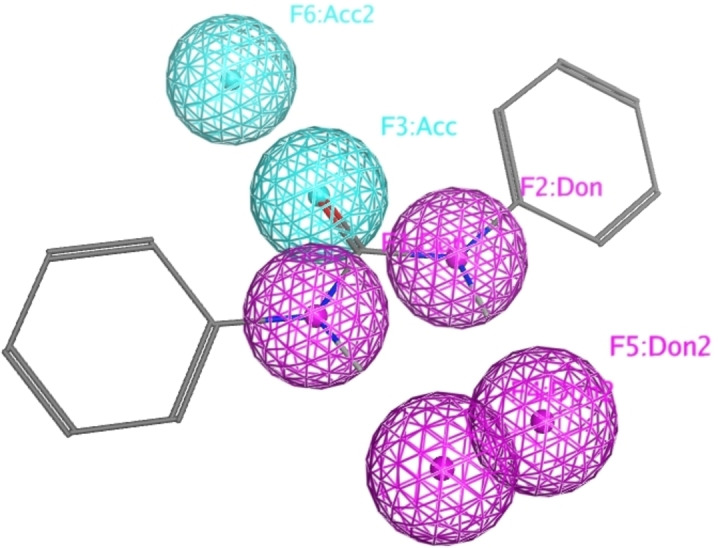
Pharmacophore of *N*,*N*’–biphenylurea modelled using Molecular Operating Environment.

Modelling studies were performed using the Molecular Operating Environment (MOE) software suite. A simple pharmacophore model consisting of two hydrogen bond donors and one hydrogen bond acceptor in a specific geometry derived from *N*,*N*’‐biphenylurea was generated (Figure 6). This key pharmacophore should be considered in all newly designed compounds and the spatial distribution of the hydrogen bond donors/acceptors kept intact.

**Figure 7:**
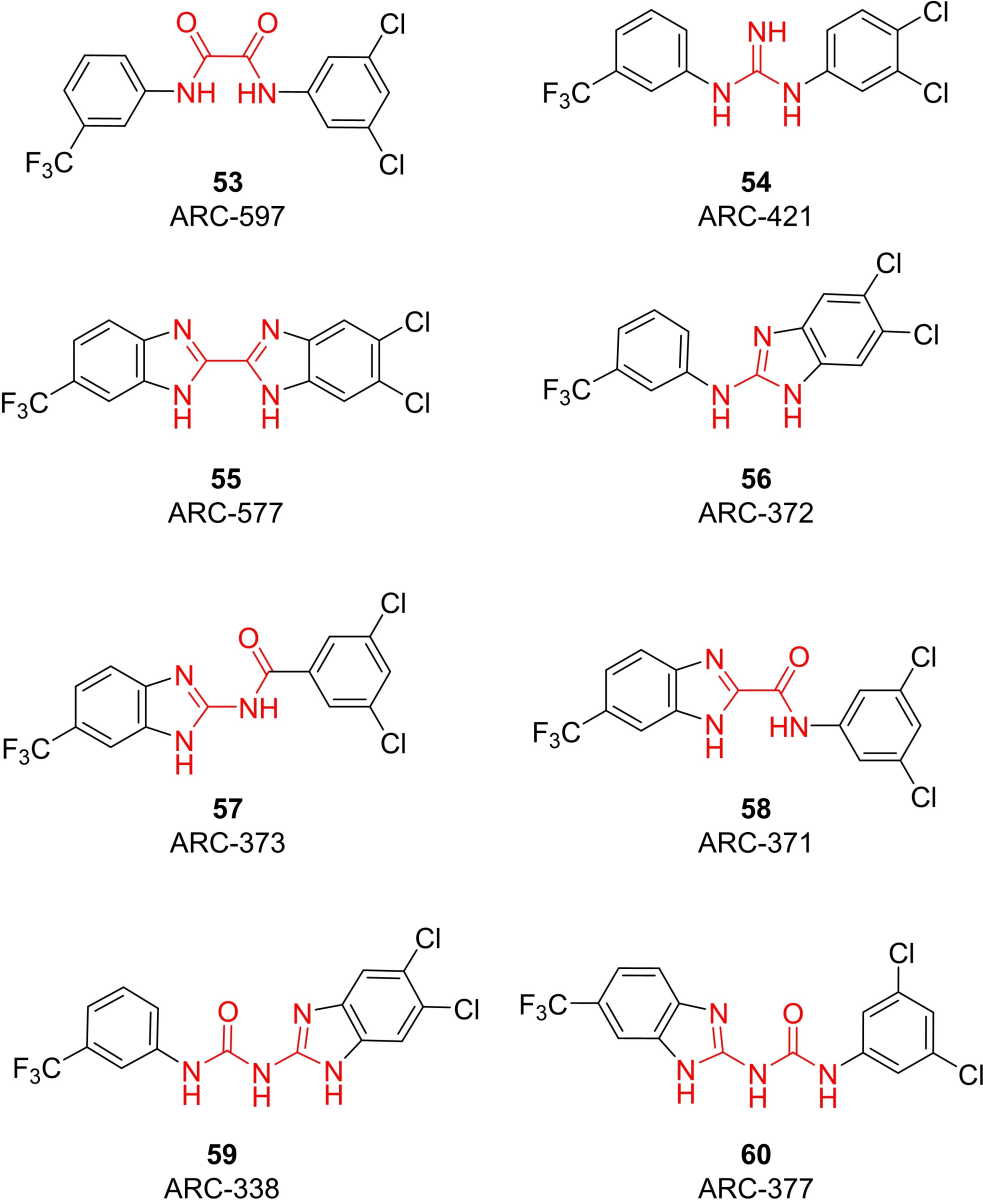
Designed novel compounds **53**–**60** (the second series).

The search of novel compouds based on the above described pharmacophore (Figure 6) was conducted on a subset of compounds from the ZINC library. Specifically, compounds with a molar mass below 200 Da and a logP value between −1 and 5 were downloaded. This initial library, totalling 1.3 million compounds, was then subjected to the pharmacophore filter to give several molecules containing groups electronically and geometrically similar to the urea motif. From these molecules, urea‐mimicking moieties were extracted, considering factors such as synthesis feasibility, biological stability, and geometric properties. Thus, new compounds **53**–**60** (Figure 7) were designed and synthesized based on the scaffold of compound **47** (Table 6) where the urea moiety was replaced with other structural motifs.

**Scheme 7:**
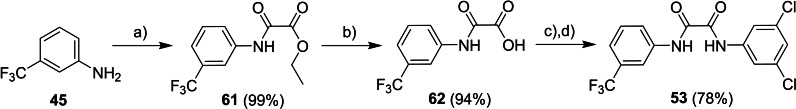
Synthesis of compound **53**. Reagents and conditions: a) diethyl oxalate, toluene, 100 °C; b) LiOH.(H_2_O)_2_, H_2_O/dioxane; c) SOCl_2_, RT; d) 3,5‐dichloroaniline, Et_3_N, DCM, RT.

Oxalyl diamide mimic **53** (Scheme 7) was synthesized by the reaction of starting 3‐(trifluoromethyl)aniline (**45**) with diethyl oxalate (to give **61**), followed by the transformation of the second carboxylic group into acid chloride and by the addition of 3,5‐dichloroaniline.

**Scheme 8:**
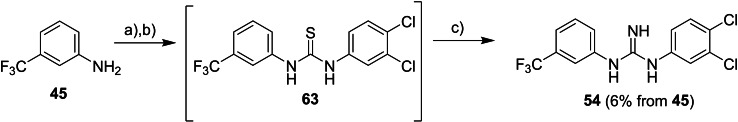
Synthesis of compound **54**. Reagents and conditions: a) TCDI, Et_3_N, RT, DCM b) 3,5‐dichloroaniline, DCM, 60 °C c) IBX, aq. NH_3_, CH_3_CN, RT.

Crude thiourea derivative **63** (prepared from aniline **45**) was transformed into guanidine derivative **54** by oxidative procedure using 2‐iodoxybenzoic acid (IBX) in the presence of aqueous ammonia (Scheme 8). The corresponding urea derivative was also formed as a side product in the last reaction step according to the UPLC‐MS spectra of the reaction mixture.

**Scheme 9:**
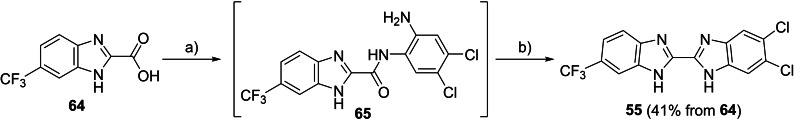
Synthesis of compound **55**. Reagents and conditions: a) 4,5‐dichlorobenzene‐1,2‐diamine, Et_3_N, HATU, DMF, RT; b) AcOH, 65 °C.

Synthesis of 2,2’‐bisimidazol derivative **55** was performed by HATU‐facilitated coupling of 5‐(trifluoromethyl)‐1*H*‐benzo[*d*]imidazole‐2‐carboxylic acid (**64**) and 4,5‐dichlorobenzene‐1,2‐diamine (to give **65**), followed by the cyclization in acetic acid (Scheme 9).

**Scheme 10:**
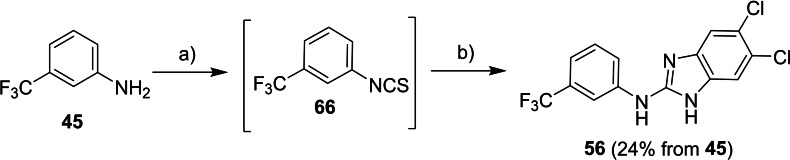
Synthesis of compound **56**. Reagents and conditions: a) TCDI, DCM, 0 °C; b) 4,5‐dichlorobenzene‐1,2‐diamine, DCM, 60 °C.

2‐Aminoimidazole derivative **56** was prepared by the *in situ* cyclization of isothiocyanate **66** which was prepared from 3‐(trifluoromethyl)aniline (**45**) by the reaction with TCDI (Scheme 10).

**Scheme 11:**
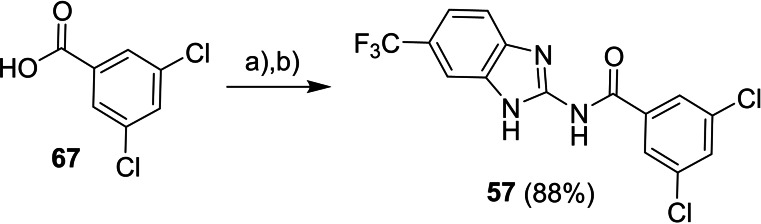
Synthesis of compound **57**. Reagents and conditions: a) SOCl_2_, RT; b) 6‐(trifluoromethyl)‐1*H*‐benzo[*d*]imidazol‐2‐amine, Et_3_N, DCM, RT.

Synthesis of compound **57** exploited a conversion of 3,5‐dichlorobenzoic acid (**67**) into its chloride which was subsequently treated with 6‐(trifluoromethyl)‐1*H*‐benzo[*d*]imidazol‐2‐amine (Scheme 11).

**Scheme 12:**
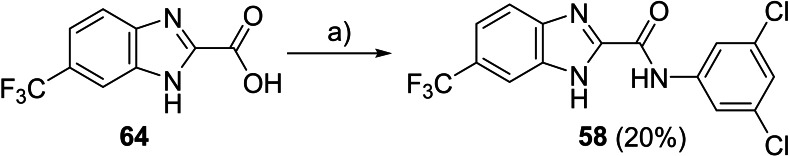
Synthesis of compound **58**. Reagents and conditions: a) 3,5‐dichloroaniline, HBTU, Et_3_N, RT.

Compound **58** was prepared by the coupling of carboxylic acid **64** with 3,5‐dichloroaniline and HBTU (Scheme 12).

**Scheme 13:**
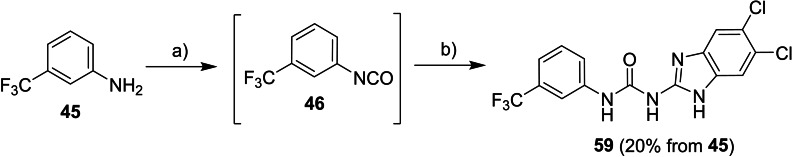
Synthesis of compound **59**. Reagents and conditions: a) triphosgene, Et_3_N, DCM, RT; b) 5,6‐dichloro‐1*H*‐benzo[d]imidazol‐2‐amine, DCM, 60 °C.

Urea derivative **59** was synthesized by the treatment of the above mentioned isocyanatobenzene **46** (prepared from **45**) with 5,6‐dichloro‐1*H*‐benzo[d]imidazol‐2‐amine (Scheme 13).

**Scheme 14:**
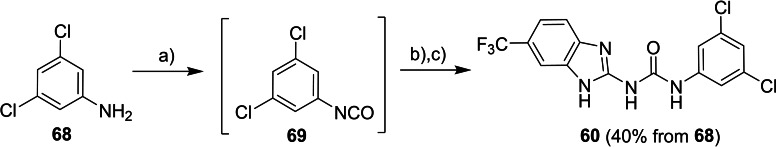
Synthesis of compound **60**. Reagents and conditions: a) triphosgene, Et_3_N, DCM, RT; b) tert‐butyl 2‐amino‐6‐(trifluoromethyl)‐1*H*‐benzo[d]imidazole‐1‐carboxylate, DCM, 60 °C; c) TFA, RT.

Analogously, urea derivative **60** was prepared by the treatment of isocyanatobenzene **69** (prepared from aniline **68**) with Boc‐protected 6‐(trifluoromethyl)‐1*H*‐benzo[d]imidazol‐2‐amine, followed by the Boc group removal (Scheme 14).

### Biological Activity of the Second Series of Compounds

Evaluation of the second series of compounds by using the enzymatic assay (Table 8) revealed that the active isosteres of urea are compounds containing 2,2’‐bisimidazole (**55**), 2‐aminoimidazole (**56**), (1*H*‐imidazol‐2‐yl)‐urea (**59, 60**). This demonstrates that urea or geometrically very close scaffolds are important for the preservation of compounds potency. Compound **60** (RA=28 %) was the most potent compound of both series.

**Table 8:** Activity of SARS‐CoV‐2 RdRp complex in the presence of compounds **53**–**60** (the 2^nd^ series derived from compound **47**) shown as residual activity (RA) in %.^[a]^

Compd	Structure	RA (%)
**53**	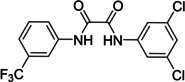	**100**
**54**	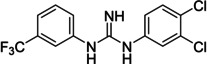	**100**
**55**	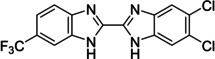	**42**
**56**	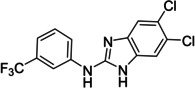	**60**
**57**	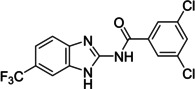	**100**
**58**	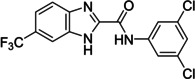	**100**
**59**	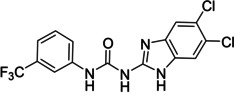	**50**
**60**	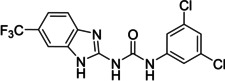	**28**

[a] RA (%) is the residual activity of the SARS‐CoV‐2 RdRp complex.

Testing of Calu‐3 cells infected with SARS‐CoV‐2 (Table 9) revealed that the compounds either retained the cytotoxicity (poor selectivity index) or lost the antiviral activity almost completely (compounds **56** and **59**). An interesting phenomenon was observed for structurally similar compounds **59** and **60**. Although these compounds differ only by the position of the inserted imidazole moiety, compound **60** is more potent as well as toxic. This finding implied that the binding site of the ligand is not symmetrical.

**Table 9:** EC_50_ and CC_50_ of the 2^nd^ series of derivatives in Calu‐3 cells infected with SARS‐CoV‐2.

Compd	EC_50_ [μM]	CC_50_ [μM]
**47**	>0.49	0.49
**55**	>2.1	2.1
**56**	>43	43
**59**	~10	23
**60**	>0.65	0.65
Remdesivir	0.11	>5

Kinetic solubility tests were performed in the medium used for the enzymatic assay–aqueous solution of HEPES buffer with 5 % DMSO. The solubility of all inhibitory compounds of the 2nd series was lower than the screening concentration, i. e., 10 μM (Table 10). This poor aqueous solubility represents a big hurdle in the development of potential antivirals and the improvement of solubility will be considered in further structural optimization efforts of this class of compounds.

**Table 10:** Kinetic solubility of selected compounds from the second series in HEPES buffer with 5 % DMSO.^[a]^

Compd	Screening 10 μM (RA %)	Kinetic solubility (μM)
**47**	45	<2.0
**55**	42	2.9±0.6
**56**	60	7.0±0.1
**59**	50	2.53±0.03
**60**	28	<2.1

[a] RA (%) is the residual activity of the SARS‐CoV‐2 RdRp complex.

Furthermore, compound **60** contains three hydrogen bond donors in a row which does not seem to be a promising drug‐like motif. Thus, compound **47** and slightly more soluble compound **55** represent better starting points for future research of potential antiviral agents based on allosteric inhibition of SARS‐CoV‐2 RNA‐dependent RNA polymerase.

## Conclusions

The global pandemic of COVID‐19 disease, caused by the highly contagious and deadly SARS‐CoV‐2, has been partially addressed by the approval of a few small‐molecule drugs, including molnupiravir and remdesivir. However, these drugs targeting the catalytic site of viral RNA‐dependent RNA polymerase (RdRp) have limitations. These include the potential development of resistant viral variants and limited efficacy in clinical settings. This highlights the potential advantages of investigating allosteric site inhibitors that could enhance the efficacy of existing treatments in a synergistic manner.

This work was focused on the development of allosteric inhibitors. Virtual docking was employed to identify several potential candidates that bind to an allosteric pocket of RdRp. Notably, compound **1** with *N*,*N*’‐diphenylurea scaffold showed moderate inhibitory activity (RA=57 %) and enhanced the efficacy of remdesivir. Further structure‐activity relationship (SAR) study led to the discovery of potent compound **47**, which despite its high cytotoxicity in Calu‐3 cells, demonstrated promising inhibitory activity (RA=45 %).

In subsequent SAR studies, we developed analogues based on *N*,*N*’‐diphenylurea isosters including 2,2’‐bisimidazol (compound **55** with RA=42 %) and on (1*H*‐imidazol‐2‐yl)urea (compounds **59** and **60**, with RA=50 and 28 %, respectively). Although these compounds retain a certain level of cytotoxicity in Calu‐3 cells, they exhibit potent inhibition of RdRp activity.

The most promising compounds represent an excellent starting point and valuable leads for further structural optimization, which will refine these potential allosteric inhibitors of SARS‐CoV‐2 RdRp. The primary objective is to reduce their cytotoxicity and improve aqueous solubility, thereby enhancing their potential as effective allosteric inhibitors of SARS‐CoV‐2 RdRp.

## Experimental Part

### General Methods

Unless otherwise stated, solvents were evaporated at 40 °C/2 kPa and prepared compounds were dried at 30 °C at 2 kPa. Starting compounds and reagents were purchased from commercial suppliers (Sigma‐Aldrich, Fluorochem, Acros Organics, TCI, AmBeed, and Vitas‐M Laboratory) and used without further purification or were prepared according to the published procedures. Tetrahydrofuran (THF), dioxane, and acetonitrile were dried by activated neutral alumina (drysphere®). Dimethylformamide (DMF) was dried by activated molecular sieves (3 Å). Other dry solvents were purchased from commercial suppliers (Sigma‐Aldrich and Acros Organics). Triethylamine was dried over potassium hydroxide under an argon atmosphere in dark flask sealed with septum.

Flash column chromatography was carried out by Teledyne ISCO Grace with dual absorbance detector on Teledyne ISCO columns RediSepRf HP C18 Aq GOLD in sizes 50 g and 100 g. Eluents used were methanol, acetonitrile, and water. Preparative LC purifications were performed on Waters Delta 600 chromatography system with columns packed with C18 reversed phase resin–Phenomenex Gemini 10 μm 21×250 mm. Mass spectra, UV absorbance, and purity of compounds were measured on Waters UPLC‐MS system consisting of Waters UPLC H‐Class Core System (column Waters Cortecs UPLC C18 1.6 μm, 2.1×50 mm), Waters Acquity UPLC PDA detector, and Mass spectrometer Waters SQD2. Yields were determined based on the amount of isolated compound. All final compounds were >95 % pure by HPLC analysis. The universal LC method was used (eluent H_2_O/CH_3_CN with 0.1 % of formic acid in both mobile phases, gradient 0–100 %, run length 3.5 min) and MS method (ESI+ and/or ESI− cone voltage=30 V, mass detector range 100–1000 Da). NMR spectra were recorded on Bruker Avance III HD spectrometers (^1^H at 400, 500 or 600 MHz) in DMSO‐d_6_, CDCl_3_ or D_2_O using a solvent signal as a reference (DMSO‐d_6_: 2.50 and 39.52, CDCl_3_: 7.26 and 77.00 for ^1^H and ^13^C, respectively). All structures were confirmed, and ^1^H and ^13^C signals were assigned by combining 1D and 2D NMR (H,C‐HSQC, H,C‐HMBC) experiments. High‐resolution mass spectra were measured on a LTQ Orbitrap XL spectrometer (Thermo Fisher Scientific).

### General Procedure for Synthesis of Isocyanates (Method A)

The corresponding aniline (1.0 equiv) was dissolved in a mixture of DCM (30 mL) and Et_3_N (3.0 equiv), and the mixture was added dropwise to the ice‐cold solution of triphosgene (1.0 equiv) in DCM (30 mL) over 40 min. The reaction was completed in 30 min after the addition. Solvents were removed under reduced pressure to give the crude product. This crude mixture (estimated aliquot amount of isocyanate) was used in the subsequent reaction without further purification due to the high reactivity of the compound.

### General Procedure for Synthesis of Substituted N,N’‐Diphenylurea Derivatives via Coupling of Anilines with Isocyanates (Method B)

The corresponding aniline (1.0 equiv) was dissolved in DCM (10 mL) and isocyanate mixture (from Method A) was added (~1.0 equiv. of isocyanate). The reaction mixture was heated to 60 °C for 12 h. Solvent was removed under reduced pressure, the crude product was dissolved in DMF, applied on flash chromatography column and separated (RP−C18aq, eluent water/methanol, gradient 0–100 %) to obtain the final product.

### Chemistry–Synthesis of Compounds

#### Dimethyl 5‐Aminoisophthalate (3)


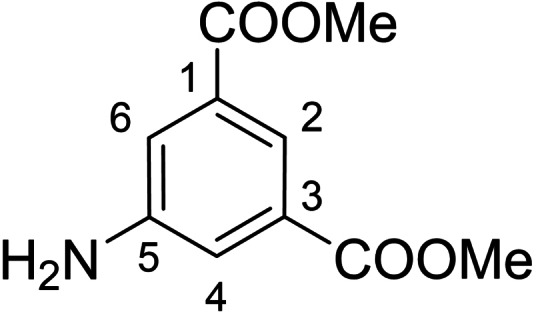


Thionyl chloride (4 mL, 55.2 mmol, 2.0 equiv) was added to methanol (150 mL) at 0 °C and after 5 min, 5‐aminoisophtalic acid (5 g, 27.6 mmol, 1.0 equiv) was added. The reaction was heated to 65 °C overnight. Solvent was evaporated and the residue was separated between aq. NaHCO_3_ saturated solution and DCM. The aqueous phase was extracted three times with DCM and the combined organic phases were extracted with aq. NaCl saturated solution. Combined organic phases were dried over CaSO_4_, drying agent was removed and solvent was evaporated to give **3** (5.34 g, 93 %) as a yellowish solid. NMR spectral data correspond with the literature. MS (ESI+): *m/z* [M+H]^+^ calculated for C_10_H_12_O_4_N=210.1, found: 210.2.

#### Dimethyl 5‐Isocyanatoisophthalate (4)


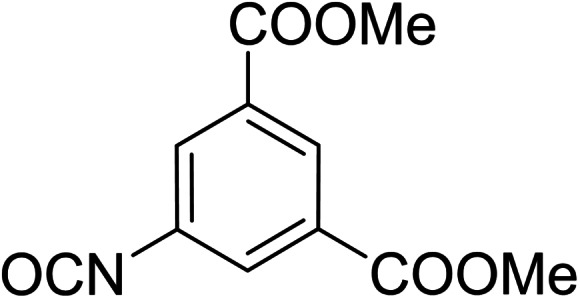


Compound **4** was synthesized from dimethyl 5‐aminoisophthalate (320 mg, 1.53 mmol, 1.0 equiv) according to Method A. The crude product (2.1 g, conversion to isocyanate 66 %, 22 % content of **4**) was used without further purifications in the next reaction step (Method B).

#### Dimethyl 5‐(3‐Butylureido)isophthalate (5)


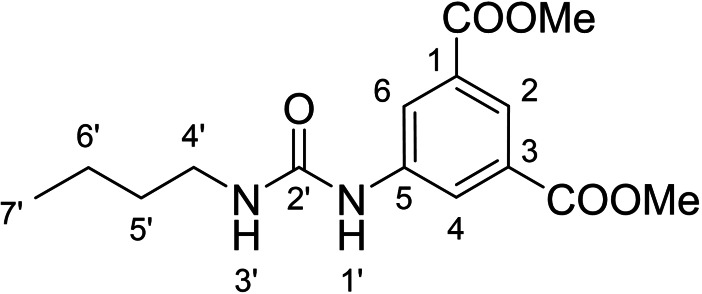


The treatment of **4** (~50 mg (227 mg of crude mixture), 0.21 mmol, 1.0 equiv) and butylamine (78 mg, 1.1 mmol, 5.0 equiv) according to Method B afforded **5** (36 mg, 55 %) as a white solid. ^1^H NMR (401 MHz, CDCl_3_) δ 8.25 (t, *J*=1.5 Hz, 1H, H2), 8.19 (d, *J*=1.6 Hz, 2H, H4, H6), 7.42 (s, 1H, H1′), 5.39 (t, *J*=5.7 Hz, 1H, H3′), 3.87 (s, 6H, OCH_3_), 3.33–3.17 (m, 2H, H4′), 1.55–1.43 (m, 2H, H5′), 1.41–1.28 (m, 2H, H6′), 0.90 (t, *J*=7.3 Hz, 3H, H7′). ^13^C NMR (101 MHz, CDCl_3_) δ 166.40 (COO), 155.70 (C2′), 140.06 (C5), 131.27 (C1, C3), 124.80 (C2), 124.39 (C4, C6), 52.54 (OCH_3_), 40.23 (C4′), 32.29 (C5′), 20.18 (C6′), 13.88 (C7′). HRMS (ESI+): *m/z* [M+H]^+^ calculated for C_15_H_21_O_5_N_2_=309.1445, found: 309.1442.

#### Dimethyl 5‐(3‐(3‐Cyanophenyl)ureido)isophthalate (6)


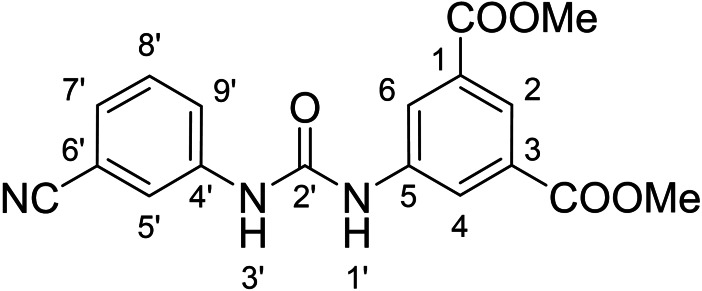


The treatment of **4** (~50 mg (227 mg of crude mixture), 0.21 mmol, 1.0 equiv) and 3‐cyanoaniline (25 mg, 0.21 mmol, 1.0 equiv) according to Method B afforded **6** (25 mg, 33 %) as a white solid. ^1^H NMR (DMSO, 401 MHz) δ 9.44 (1H, s, H1’), 9.15 (1H, s, H3’), 8.34 (2H, d, *J*=1.6 Hz, H4, H6), 8.10 (1H, t, *J*=1.6 Hz, H2), 7.99 (1H, t, *J*=1.8 Hz, H5’), 7.74–7.69 (1H, m, H9’), 7.53–7.48 (1H, m, H8’), 7.46–7.43 (1H, m, H7’), 3.89 (6H, s, OCH_3_). ^13^C NMR (DMSO, 101 MHz) δ 165.36 (COO), 152.43 (C2’), 140.52 (C5), 140.29 (C4’), 130.70 (C1, C‐3), 130.18 (C8’), 125.73 (C7’), 123.34 (C2), 122.97 (C9’), 122.93 (C4, C6), 121.26 (C5’), 118.82 (CN), 111.61 (C6’), 52.54 (OCH_3_). HRMS (ESI+): *m/z* [M+H]^+^ calculated for C_18_H_16_O_5_N_3_=354.1085, found: 354.1084.

#### Dimethyl 5‐(3‐(4‐Cyanophenyl)ureido)isophthalate (7)


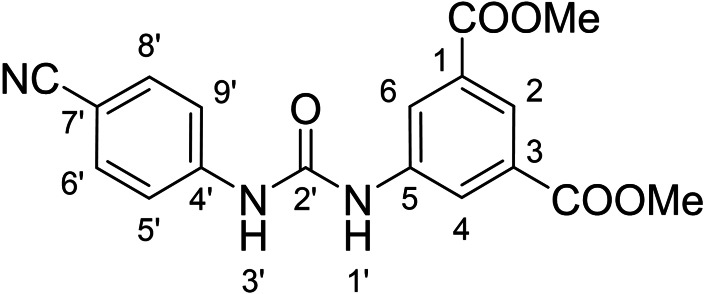


The treatment of **4** (~70 mg (318 mg of crude mixture), 0.30 mmol, 1.0 equiv) and 4‐cyanoaniline (35 mg, 0.30 mmol, 1.0 equiv) according to Method B afforded **7** (23 mg, 22 %) as a white solid. ^1^H NMR (DMSO, 401 MHz) δ 9.40 (2H, s, H‐1’, H‐3’), 8.34 (2H, q, *J*=1.2 Hz, H‐4, H‐6), 8.11 (1H, p, *J*=1.3 Hz, H‐2), 7.74 (2H, d, *J*=8.8 Hz, H‐5’, H‐9’), 7.71–7.63 (2H, m, H‐6’, H‐8’), 3.89 (6H, s, OCH_3_). ^13^C NMR (DMSO, 101 MHz) δ 165.34 (COO), 152.15 (C‐2’), 143.88 (C‐4’), 140.38 (C‐5), 133.27 (C‐5’, C‐9’), 130.72 (C‐1, C‐3), 123.09 (C‐2), 123.02 (C‐4, C‐6), 119.25 (CN), 118.47 (C‐6’, C‐8’), 103.68 (C‐7’), 52.55 (OCH_3_). HRMS (ESI+): *m/z* [M+H]^+^ calculated for C_18_H_16_O_5_N_3_=354.1085, found: 354.1083.

#### Dimethyl 5‐(3‐(4‐Sulfamoylphenyl)ureido)isophthalate (8)


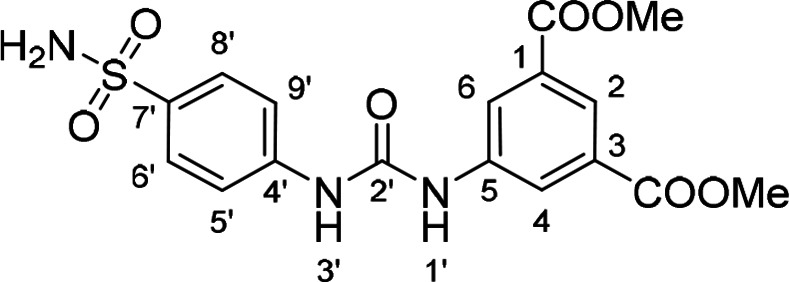


The treatment of **4** (~70 mg (318 mg of crude mixture), 0.30 mmol, 1.0 equiv) and sulfanilamide (51 mg, 0.30 mmol, 1.0 equiv) according to Method B afforded **8** (42 mg, 35 %) as a white solid. ^1^H NMR (DMSO, 401 MHz) δ 9.40 (1H, s, H‐1’), 9.21 (1H, s, H‐3’), 8.35 (2H, d, *J*=1.5 Hz, H‐4, H‐6), 8.11 (1H, t, *J*=1.5 Hz, H‐2), 7.75 (2H, d, *J*=8.9 Hz, H‐6’, H‐8’), 7.65 (2H, d, *J*=8.9 Hz, H‐5’, H‐9’), 7.22 (2H, s, NH_2_), 3.90 (6H, s, OCH_3_). ^13^C NMR (DMSO, 101 MHz) δ 165.36 (COO), 152.28 (C‐2’), 142.46 (C‐4’), 140.54 (C‐5), 137.26 (C‐7’), 130.72 (C‐1, C‐3), 126.80 (C‐6’, C‐8’), 122.95 (C‐2), 122.88 (C‐4, C‐6), 117.90 (C‐5’, C‐9’), 52.55 (OCH_3_). HRMS (ESI+): *m/z* [M+H]^+^ calculated for C_17_H_18_O_7_N_3_S=408.0860, found: 408.0861.

#### Dimethyl 5‐(3‐(3‐Trifluoromethylphenyl)ureido)isophthalate (24)


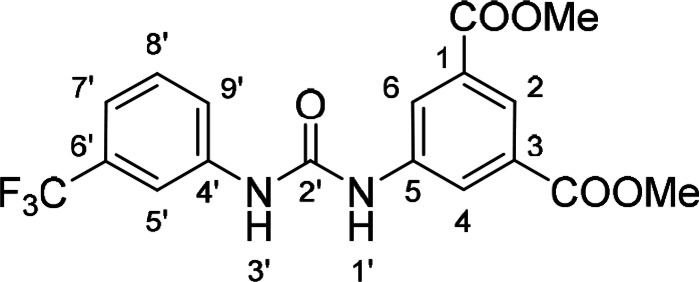


The treatment of **4** (~70 mg (318 mg of crude mixture), 0.30 mmol, 1.0 equiv) and 3‐trifluoroaniline (53 mg, 0.33 mmol, 1.1 equiv) according to Method B afforded **24** (70 mg, 59 %) as a white solid. ^1^H NMR (DMSO, 401 MHz) δ 9.39 (1H, s, H‐1’), 9.16 (1H, s, H‐3’), 8.36 (2H, d, *J*=1.6 Hz, H‐4, H‐6), 8.12 (1H, t, *J*=1.6 Hz, H‐2), 8.02 (1H, s, H‐5’), 7.68–7.61 (1H, m, H‐9’), 7.54 (1H, dd, *J*=7.9, 7.9 Hz, H‐8’), 7.35 (1H, d, *J*=7.7 Hz, H‐7’), 3.90 (6H, s, OCH_3_). ^13^C NMR (DMSO, 101 MHz) δ 165.85 (COO), 152.99 (C‐2’), 141.05 (C‐5), 140.70 (C‐4’), 131.17 (C‐1, C‐3), 130.40 (C‐8’), 123.43 (C‐4, C‐6), 123.40 (C‐2), 122.80 (C‐9’), 119.00 (C‐7’), 115.05 (d, *J*=4.4 Hz, C‐5’), 53.01 (OCH_3_). HRMS (ESI+): *m/z* [M+H]^+^ calculated for C_18_H_16_O_5_N_2_F_3_=397.1006, found: 397.1003.

#### Dimethyl 5‐(3‐(3‐(Ethoxycarbonyl)phenyl)ureido)isophthalate (10)


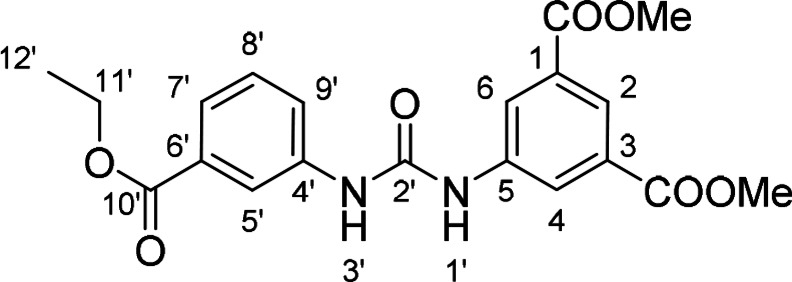


The treatment of **4** (~70 mg (318 mg of crude mixture), 0.30 mmol, 1.0 equiv) and ethyl 3‐aminobenzoate (54 mg, 0.33 mmol, 1.1 equiv) according to Method B afforded **10** (63 mg, 53 %) as a white solid. ^1^H NMR (DMSO, 401 MHz) δ 9.27 (1H, s, H‐1’), 9.05 (1H, s, H‐3’), 8.35 (2H, d, *J*=1.6 Hz, H‐4, H‐6), 8.17 (1H, t, *J*=1.9 Hz, H‐5’), 8.10 (1H, t, *J*=1.6 Hz, H‐2), 7.69 (1H, ddd, *J*=8.1, 2.3, 1.1 Hz, H‐9’), 7.60 (1H, ddd, *J*=7.7, 1.3, 1.3 Hz, H‐7’), 7.44 (1H, dd, *J*=7.9, 7.9 Hz, H‐8’), 4.33 (2H, q, *J*=7.1 Hz, H‐11’), 3.90 (6H, s, OCH_3_), 1.33 (3H, t, *J*=7.1 Hz, H‐12’). ^13^C NMR (DMSO, 101 MHz) δ 165.67 (C‐10’), 165.39 (**C**OOMe), 152.50 (C‐2’), 140.70 (C‐5), 139.75 (C‐4’), 130.68 (C‐1, C‐3), 130.45 (C‐6’), 129.19 (C‐8’), 123.20 (C‐9’), 122.88 (C‐4, C‐6, C‐7’), 122.81 (C‐2), 119.01 (C‐5’), 60.79 (C‐11’), 52.53 (OCH_3_), 14.21 (C‐12’). HRMS (ESI+): *m/z* [M+H]^+^ calculated for C_20_H_21_O_7_N_2_=401.1343, found: 401.1344.

#### Dimethyl 5‐(3‐(3,5‐Bis(trifluoromethyl)phenyl)ureido)isophthalate (11)


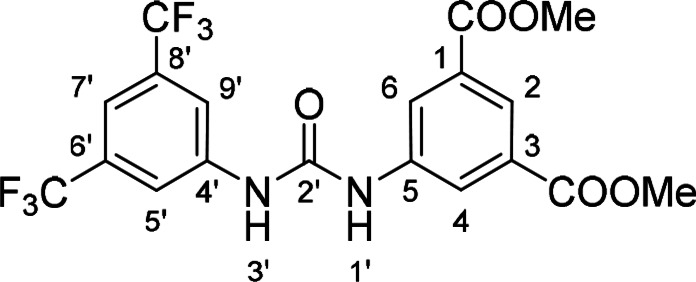


The treatment of **4** (~50 mg (227 mg of the crude mixture), 0.21 mmol, 1.0 equiv) and 3,5‐bis(trifluoromethyl)aniline (49 mg, 0.21 mmol, 1.0 equiv) according to Method B afforded **11** (66 mg, 67 %) as a white solid. ^1^H NMR (DMSO, 401 MHz) δ 9.58 (1H, s, H‐1’), 9.51 (1H, s, H‐3’), 8.38 (2H, d, *J*=1.6 Hz, H‐4, H‐6), 8.18 (2H, d, *J*=1.6 Hz, H‐5’, H‐9’), 8.13 (1H, t, *J*=1.6 Hz, H‐2), 7.68 (1H, s, H‐7’), 3.90 (6H, s, OCH_3_). ^13^C NMR (DMSO, 101 MHz) δ 165.33 (COO), 152.48 (C‐2’), 141.55 (C‐4’), 140.30 (C‐5), 130.70 (C‐1, C‐3), 130.69 (d, *J*=32.7 Hz, C‐6’, C‐8’), 123.32 (C‐4, C‐6), 123.31 (d, *J*=272.6 Hz, CF_3_), 123.26 (C‐2), 118.48 (C‐5’, C‐9’), 114.83 (C‐7’), 52.55 (OCH_3_). HRMS (ESI+): *m/z* [M+H]^+^ calculated for C_19_H_15_O_5_N_2_F_6_=465.0880, found: 465.0878.

#### Dimethyl 5‐(3‐(3,5‐Dinitophenyl)ureido)isophthalate (12)


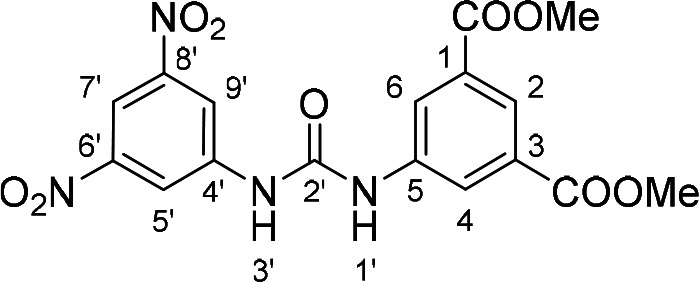


The treatment of **4** (~50 mg (227 mg of the crude mixture), 0.21 mmol, 1.0 equiv) and 3,5‐dinitroaniline (39 mg, 0.21 mmol, 1.0 equiv) according to the Method B afforded **12** (35 mg, 39 %) as a yellowish solid. ^1^H NMR (DMSO, 401 MHz) δ 9.83 (1H, s, H‐3’), 9.67 (1H, s, H‐1’), 8.79 (2H, d, *J*=2.1 Hz, H‐5’, H‐9’), 8.43 (1H, t, *J*=2.1 Hz, H‐7’), 8.40 (2H, d, *J*=1.6 Hz, H‐4, H‐6), 8.14 (1H, t, *J*=1.6 Hz, H‐2), 3.91 (6H, s, OCH_3_). ^13^C NMR (DMSO, 101 MHz) δ 165.31 (COO), 152.36 (C‐2’), 148.24 (C‐6’, C‐8’), 141.94 (C‐4’), 140.15 (C‐5), 130.71 (C‐1, C‐3), 123.49 (C‐4, C‐6), 123.43 (C‐2), 118.10 (C‐5’, C‐9’), 111.07 (C‐7’), 52.57 (OCH_3_). HRMS (ESI+): *m/z* [M+H]^+^ calculated for C_17_H_15_O_9_N_4_=419.0834, found: 419.0835.

#### 3‐(3‐(3,5‐Bis(methoxycarbonyl)phenyl)ureido)benzoic Acid (13)


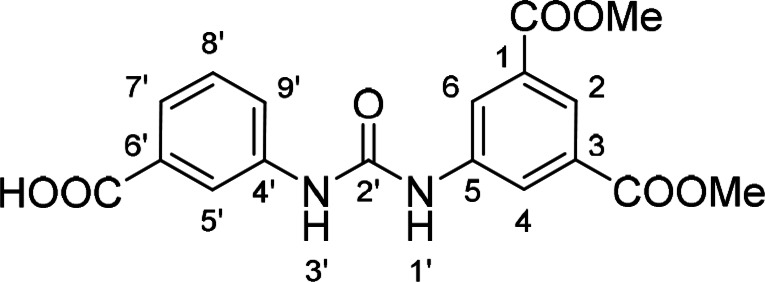


The treatment of **4** (~50 mg (227 mg of the crude mixture), 0.21 mmol, 1.0 equiv) and 3‐aminobenzoic acid (29 mg, 0.21 mmol, 1.0 equiv) according to the Method B afforded **13** (43 mg, 54 %) as a beige solid. ^1^H NMR (DMSO, 401 MHz) δ 12.95 (COO**H**), 9.29 (1H, s, H‐1’), 9.00 (1H, s, H‐3’), 8.36 (2H, d, *J*=1.5 Hz, H‐4, H‐6), 8.16 (1H, t, *J*=1.9 Hz, H‐5’), 8.10 (1H, t, *J*=1.6 Hz, H‐2), 7.69–7.64 (1H, m, H‐9’), 7.58 (1H, dt, *J*=7.7, 1.4 Hz, H‐7’), 7.42 (1H, t, *J*=7.9 Hz, H‐8’), 3.90 (6H, s, OCH_3_). ^13^C NMR (DMSO, 101 MHz) δ 167.27 (**C**OOH), 165.40 (**C**OOMe), 152.51 (C‐2’), 140.76 (C‐5), 139.60 (C‐4’), 131.38 (C‐6’), 130.69 (C‐1, C‐3), 129.00 (C‐8’), 123.09 (C‐7’), 122.85 (C‐9’), 122.83 (C‐4, C‐6), 122.76 (C‐2), 119.33 (C‐5’), 52.53 (OCH_3_). HRMS (ESI+): *m/z* [M+H]^+^ calculated for C_18_H_17_O_7_N_2_=373.1030, found: 373.1031.

#### Dimethyl 5‐(3‐(Pyridin‐4‐yl)ureido)isophthalate (14)


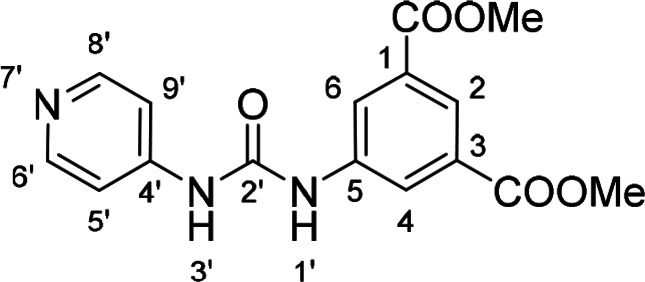


The treatment of **4** (~50 mg (227 mg of the crude mixture), 0.21 mmol, 1.0 equiv) and 3‐aminobenzoic acid (20 mg, 0.21 mmol, 1.0 equiv) according to the Method B afforded **14** (42 mg, 60 %) as a yellowish solid. ^1^H NMR (DMSO, 401 MHz) δ 9.67 (1H, s, H‐1’), 9.62 (1H, s, H‐3’), 8.45–8.39 (2H, m, H‐6’, H‐8’), 8.34 (2H, d, *J*=1.6 Hz, H‐4, H‐6), 8.12 (1H, t, *J*=1.6 Hz, H‐2), 7.56–7.53 (2H, m, H‐5’, H‐9’), 3.90 (6H, s, OCH_3_). ^13^C NMR (DMSO, 101 MHz) δ 165.30 (COO), 152.03 (C‐2’), 148.78 (C‐6’, C‐8’), 147.50 (C‐4’), 140.16 (C‐5), 130.76 (C‐1, C‐3), 123.02 (C‐4, C‐6), 122.97 (C‐2), 112.67 (C‐5’, C‐9’), 52.56 (OCH_3_). HRMS (ESI+): *m/z* [M+H]^+^ calculated for C_16_H_16_O_5_N_3_=330.1085, found: 330.1085.

#### 1‐Isocyanato‐3‐Nitrobenzene (16)


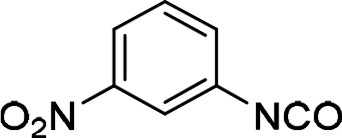


Compound **16** was synthesized from dimethyl 3‐nitroaniline (5.1 g, 37 mmol, 1.0 equiv) according to the Method A. The crude product (14.1 g, conversion to isocyanate 67 %, 29 % content of **16**) was used without further purifications in the next reaction step (Method B).

#### Dimethyl 5‐(3‐(3‐Nitrophenyl)ureido)isophthalate (17)


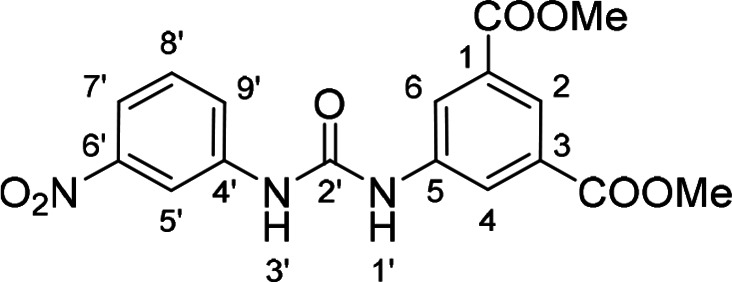


The treatment of **16** (~217 mg (748 mg of the crude mixture), 1.3 mmol, 1.0 equiv) and **3** (276 mg, 1.3 mmol, 1.0 equiv) according to Method B afforded **17** (50 mg, 10 %) as a yellowish solid. ^1^H NMR (DMSO, 401 MHz) δ 9.41 (1H, s, H‐3’), 9.32 (1H, s, H‐1’), 8.56 (1H, dd, *J*=2.2, 2.2 Hz, H‐5’), 8.36 (2H, d, *J*=1.5 Hz, H‐4, H‐6), 8.11 (1H, d, *J*=1.5 Hz, H‐2), 7.88–7.83 (1H, m, H‐7’), 7.80–7.75 (1H, m, H‐9’), 7.62–7.55 (1H, m, H‐8’), 3.90 (6H, s, OCH_3_). ^13^C NMR (DMSO, 101 MHz) δ 165.35 (COO), 152.43 (C‐2’), 140.45 (C‐4’), 138.58 (C‐5), 130.69 (C‐1, C‐3), 130.07 (C‐8’), 124.75 (C‐2), 123.04 (C‐4, C‐6), 116.74 (C‐7’), 116.69 (C‐9’), 112.57 (C‐5’), 52.54 (OCH_3_). MS (ESI+): *m/z* [M+H]^+^ calculated for C_17_H_15_O_7_N_3_=374.1, found: 374.3.

#### 1,3‐Bis(3‐nitrophenyl)urea (18)


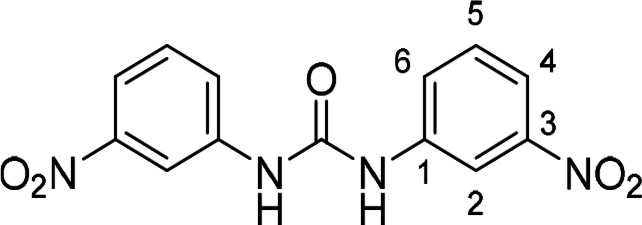


The treatment of **16** (~200 mg (690 mg of the crude mixture), 1.2 mmol, 1.0 equiv) and 3‐nitroaniline (168 mg, 1.2 mmol, 1.0 equiv) according to Method B afforded **18** (98 mg, 27 %) as a yellow solid. ^1^H NMR (DMSO, 401 MHz) δ 9.39 (2H, s, NH), 8.56 (2H, t, *J*=2.2 Hz, H‐2), 7.86 (2H, ddd, *J*=8.2, 2.3, 0.9 Hz, H‐4), 7.77 (2H, ddd, *J*=8.2, 2.2, 1.0 Hz, H‐6), 7.59 (2H, t, *J*=8.1 Hz, H‐5). ^13^C NMR (DMSO, 101 MHz) δ 152.42 (CON), 148.13 (C‐3), 140.64 (C‐1), 130.13 (C‐5), 124.71 (C‐6), 116.74 (C‐4), 112.53 (C‐2). HRMS (ESI+): *m/z* [M+H]^+^ calculated for C_13_H_11_O_5_N_4_=303.0724, found: 303.0725.

#### 1‐(3,5‐Bis(trifluoromethyl)phenyl)‐3‐(3‐Nitrophenyl)urea (19)


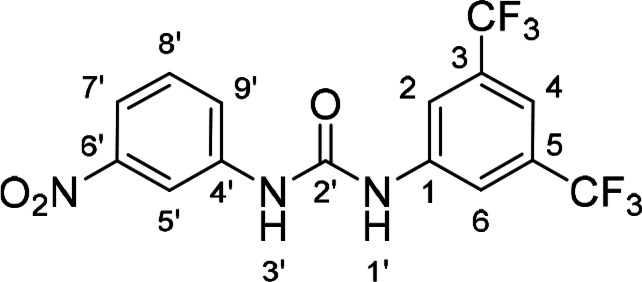


The treatment of **16** (~50 mg (172 mg of the crude mixture), 0.31 mmol, 1.0 equiv) and 3,5‐bis(trifluoromethyl)aniline (70 mg, 0.31 mmol, 1.0 equiv) according to the Method B afforded **19** (73 mg, 61 %) as a white solid. ^1^H NMR (DMSO, 401 MHz) δ 9.58 (1H, s, H‐1’), 9.56 (1H, s, H‐3’), 8.54 (1H, dd, *J*=2.2, 2.2 Hz, H‐5’), 8.16 (2H, d, *J*=1.6 Hz, H‐2, H‐6), 7.87 (1H, ddd, *J*=8.2, 2.3, 0.9 Hz, H‐7’), 7.80 (1H, ddd, *J*=8.2, 2.2, 0.9 Hz, H‐9’), 7.68 (1H, s, H‐4), 7.59 (1H, dd, *J*=8.2, 8.2 Hz, H‐8’). ^13^C NMR (DMSO, 101 MHz) δ 152.45 (C‐2’), 148.10 (C‐6’), 141.49 (C‐1), 140.46 (C‐4’), 130.73 (q, *J*=32.7 Hz, C‐3, C‐5), 130.14 (C‐8’), 124.97 (C‐9’), 123.30 (q, *J*=272.8 Hz, CF_3_), 118.41 (d, *J*=4.1 Hz, C‐2, C‐6), 116.97 (C‐7’), 114.87 (d, *J*=7.1 Hz, C‐4), 112.81 (C‐5’). HRMS (ESI+): *m/z* [M+H]^+^ calculated for C_15_H_10_O_3_N_3_F_6_=394.0621, found: 394.0622.

#### 3‐(Methoxycarbonyl)‐5‐(3‐(3‐Nitrophenyl)ureido)benzoic Acid (20) and 5‐(3‐(3‐Nitrophenyl)ureido)isophthalic acid (21)


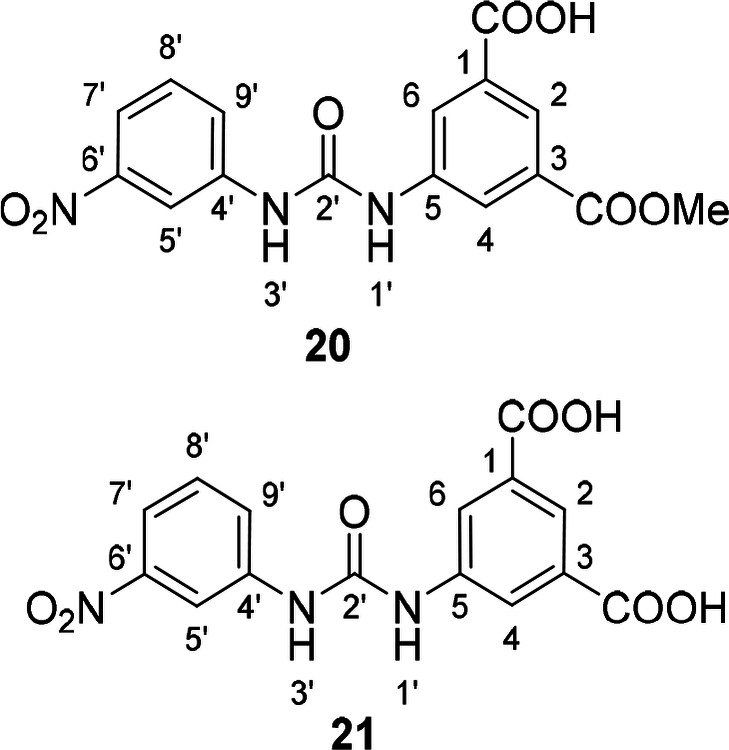


Compound **17** (40 mg, 0.11 mmol, 1.0 equiv) was dissolved in a water/dioxane (0.5 mL/0.5 mL) mixture. The solution was cooled to 0 °C and LiOH.2H_2_O (14 mg, 0.32 mmol, 3.0 equiv) was added. After 1.5 h, the starting compound was consumed (UPLC‐MS). Reaction mixture was quenched with aq. saturated NH_4_Cl solution. Solvent was removed under reduced pressure, the crude product was dissolved in DMF, applied on flash chromatography column, and separated (RP−C18aq, eluent water/methanol, gradient 0–100 %) to obtain **20** (12 mg, 31 %) as yellowish solid and **21** (16 mg, 43 %) as yellow solid.

Compound **20**: ^1^H NMR (DMSO, 401 MHz) δ 13.30 (1H, s, COO**H**), 9.47 (1H, s, H‐1’), 9.43 (1H, s, H‐3’), 8.57 (1H, dd, *J*=2.3, 2.3 Hz, H‐5’), 8.38 (1H, dd, *J*=1.9, 1.9 Hz, H‐4), 8.31–8.30 (1H, m, H‐6), 8.12 (1H, dd, *J*=1.6, 1.6 Hz, H‐2), 7.84 (1H, ddd, *J*=8.2, 2.4, 0.9 Hz, H‐7’), 7.78 (1H, ddd, *J*=8.2, 2.2, 1.0 Hz, H‐9’), 7.57 (1H, dd, *J*=8.2, 8.2 Hz, H‐8’), 3.89 (3H, s, OCH_3_). ^13^C NMR (DMSO, 101 MHz) δ 166.55 (COOH), 165.56 (COOMe), 152.51 (C‐2’), 148.12 (C‐6’), 140.83 (C‐4’), 140.30 (C‐5), 132.21 (C‐1), 130.52 (C‐3), 130.07 (C‐8’), 124.70 (C‐9’), 123.38 (C‐2, C‐6), 122.61 (C‐4), 116.60 (C‐7’), 112.52 (C‐5’), 52.47 (OCH_3_). HRMS (ESI+): *m/z* [M+H]^+^ calculated for C_16_H_14_O_7_N_3_=360.0826, found: 360.0825.

Compound **21**: ^1^H NMR (DMSO, 401 MHz) δ 13.22 (2H, s, COO**H**), 9.63–9.50 (2H, m, H‐1’, H‐3’), 8.58 (1H, dd, *J*=2.2, 2.2 Hz, H‐5’), 8.33 (2H, d, *J*=1.5 Hz, H‐4, H‐6), 8.14–8.12 (1H, m, H‐2), 7.83 (1H, dd, *J*=8.1, 2.4 Hz, H‐7’), 7.79 (1H, dd, *J*=8.1, 2.2 Hz, H‐9’), 7.57 (1H, dd, *J*=8.2, 8.2 Hz, H‐8’). ^13^C NMR (DMSO, 101 MHz) δ 166.81 (COO), 152.61 (C‐2’), 148.16 (C‐6’), 140.99 (C‐4’), 140.14 (C‐5), 132.07 (C‐1, C‐3), 130.07 (C‐8’), 124.65 (C‐9’), 123.66 (C‐2), 122.99 (C‐4, C‐6), 116.52 (C‐7’), 112.47 (C‐5’). HRMS (ESI+): *m/z* [M+H]^+^ calculated for C_15_H_12_O_7_N_3_=346.0670, found: 360.0671.

#### 1‐Isocyanato‐3‐(Trifluoromethyl)benzene (46)


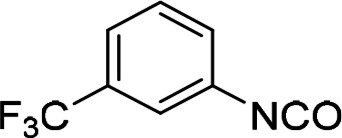


Compound **46** was synthesized from 3‐(trifluoromethyl)aniline (**45**, 2.5 g, 15.5 mmol, 1.0 equiv) according to Method A. The crude product (9.4 g, conversion to isocyanate 90 %, 28 % content of **46**) was used without further purifications in the next reaction step (Method B).

#### 1‐(3,5‐Dichlorophenyl)‐3‐(3‐(Trifluoromethyl)phenyl)urea (47)


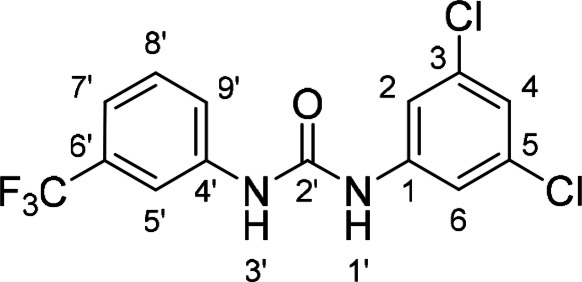


The treatment of compound **46** (~50 mg (179 mg of the crude mixture), 0.27 mmol, 1.0 equiv) and 3,5‐dichloroaniline (43 mg, 0.27 mmol, 1.0 equiv) according to the Method B afforded **47** (20 mg, 21 %) as a white solid. ^1^H NMR (DMSO, 401 MHz) δ 9.50–9.39 (2H, m, H‐1’, H‐3’), 7.98 (1H, s, H‐5’), 7.60 (1H, ddd, *J*=8.3, 1.5, 1.5 Hz, H‐9’), 7.55–7.53 (2H, m, H‐2, H‐6), 7.51 (1H, d, *J*=8.1 Hz, H‐8’), 7.36–7.31 (1H, m, H‐7’), 7.20–7.16 (1H, m, H‐4). ^13^C NMR (DMSO, 101 MHz) δ 152.30 (C‐2’), 142.00 (C‐1), 140.11 (C‐4’), 134.10 (C‐3, C‐5), 129.97 (C‐8’), 129.53 (q, *J*=31.4 Hz, C‐6’), 124.15 (q, *J*=272.2 Hz, CF_3_), 122.10 (C‐9’), 121.15 (C‐4), 118.53 (q, *J*=4.1 Hz, C‐7’), 116.43 (C‐2, C‐6), 114.37 (q, *J*=4.5 Hz, C‐5’). HRMS (ESI+): *m/z* [M+H]^+^ calculated for C_14_H_10_ON_2_Cl_2_F_3_=349.0117, found: 349.0116.

#### *N*‐(3,5‐Dichlorophenyl)‐2‐(4‐Nitrophenyl)acetamide (49)


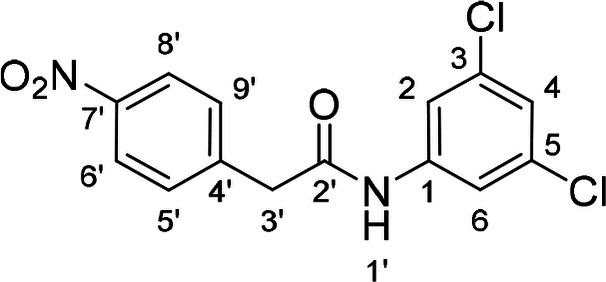


2‐(4‐Nitrophenyl)acetic acid (**48**, 100 mg, 0.55 mmol, 1.0 equiv) was dissolved in thionyl chloride (1.0 mL) and stirred for 30 min at RT. Thionyl chloride was removed on RVO, the residue was suspended in DCM and 3,5‐dichloroaniline (179 mg, 1.1 mmol, 2.0 equiv) and Et_3_N (0.6 mL, 4.4 mmol, 8.0 equiv) were added. The solution was cooled to 0 °C, LiOH.2H_2_O (145 mg, 3.45 mmol, 3.0 equiv) was added and stirred for 2 h. Solvent was removed under reduced pressure, the residue was dissolved in DMF, applied on flash chromatography column, and separated (RP−C18aq, eluent water/methanol, gradient 0–100 %) to obtain **49** (120 mg, 67 %) as a brown solid. ^1^H NMR (DMSO, 401 MHz) δ 10.65 (1H, s, H‐1’), 8.25–8.16 (2H, m, H‐6’, H‐8’), 7.66 (2H, d, *J*=1.9 Hz, H‐2, H‐6), 7.62–7.58 (2H, m, H‐5’, H‐9’), 7.28 (1H, t, *J*=1.9 Hz, H‐4), 3.87 (2H, s, H‐3’). ^13^C NMR (DMSO, 101 MHz) δ 168.79 (C‐2’), 146.47 (C‐7’), 143.27 (C‐4’), 141.26 (C‐1), 134.12 (C‐3, C‐5), 130.73 (C‐5’, C‐9’), 123.38 (C‐6’, C‐8’), 122.65 (C‐4), 117.28 (C‐2, C‐6), 42.70 (C‐3’). HRMS (ESI+): *m/z* [M+H]^+^ calculated for C_14_H_11_O_3_N_2_Cl_2_=325.0141, found: 325.0138.

#### 2‐(3,5‐Dichlorophenyl)‐*N*‐(4‐Nitrophenyl)acetamide (52)


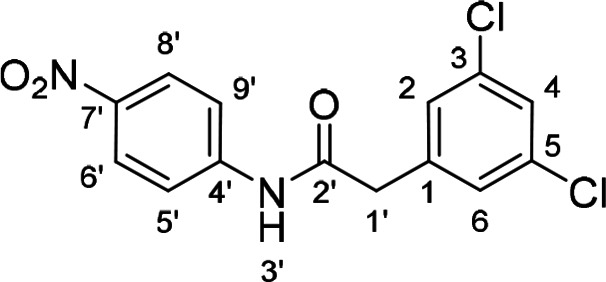


2‐(3,5‐Dichlorophenyl)acetic acid (**50**, 100 mg, 0.49 mmol, 1.0 equiv), 4‐nitroaniline (**51**, 67 mg, 0.49 mmol, 1.0 equiv), HBTU (278 mg, 0.73 mmol, 1.5 equiv) and Et_3_N (0.27 mL, 1.95 mmol, 4.0 equiv) were dissolved in dry DMF (5 mL) and stirred overnight at RT. The reaction mixture was applied on flash chromatography column and separated (RP−C18aq, eluent water/methanol, gradient 0–100 %) to obtain **52** (12 mg, 8 %) as a yellowish solid. ^1^H NMR (DMSO, 401 MHz) δ 10.80 (1H, s, H‐3’), 8.26–8.20 (1H, m, H‐6’, H‐8’), 7.87–7.81 (1H, m, H‐5’, H‐9’), 7.52 (1H, t, *J*=2.0 Hz, H‐4), 7.41 (2H, d, *J*=1.9 Hz, H‐2, H‐6), 3.80 (2H, s, H‐1’). ^13^C NMR (DMSO, 101 MHz) δ 169.09 (C‐2’), 145.12 (C‐4’), 142.27 (C‐7’), 139.29 (C‐1), 133.71 (C‐3, C‐5), 128.41 (C‐2, C‐6), 126.43 (C‐4), 125.02 (C‐6’, C‐8’), 118.86 (C‐5’, C‐9’), 42.01 (C‐1’). HRMS (ESI+): *m/z* [M+H]^+^ calculated for C_14_H_11_O_3_N_2_Cl_2_=325.0141, found: 325.0139.

#### *N* ^1^‐(3,5‐Dichlorophenyl)‐*N* ^2^‐(3‐(Trifluoromethyl)phenyl)oxalamide (53)


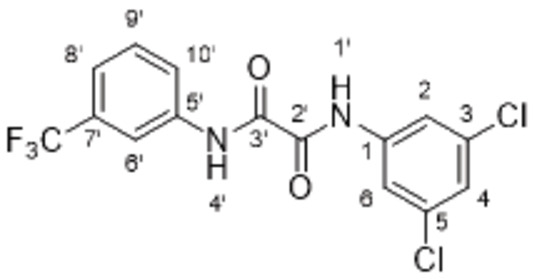


Compound **62** (30 mg, 0.13 mmol, 1.0 equiv) was dissolved in thionyl chloride (0.5 mL) and stirred at 70 °C for 1 h. Thionyl chloride was removed under reduced pressure and the liquid residue was dissolved in DCM (5.0 mL). 3,5‐Dichloroaniline (42 mg, 0.26 mmol, 2.0 equiv) and Et_3_N (0.05 mL, 0.39 mmol, 3.0 equiv) were added and the mixture was heated to 60 °C for 1 h. The mixture was removed under reduced pressure till constant volume, the residue was dissolved in DMF, applied on flash chromatography column and separated (RP−C18aq, eluent water/methanol, gradient 0–100 %) to obtain **53** (38 mg, 0.10 mmol, 78 %) as a white solid. ^1^H NMR (DMSO, 401 MHz) δ 11.22 (2H, s, H‐1’, H‐4’), 8.34–8.31 (1H, m, H‐6’), 8.16–8.11 (1H, m, H‐10’), 7.98 (2H, d, *J*=1.9 Hz, H‐2, H‐6), 7.64 (1H, dd, *J*=8.0, 8.0 Hz, H‐9’), 7.55–7.50 (1H, m, H‐8’), 7.40 (1H, t, *J*=1.9 Hz, H‐4). ^13^C NMR (DMSO, 101 MHz) δ 158.80 (C‐3’), 158.55 (C‐2’), 140.36 (C‐1), 138.45 (C‐5’), 134.06 (C‐3, C‐5), 130.06 (C‐9’), 129.46 (q, *J*=31.8 Hz, C‐7’), 124.20 (C‐10’), 124.03 (q, *J*=272.9 Hz, CF_3_), 123.84 (C‐4), 121.06 (q, *J*=4.1 Hz, C‐8’), 118.78 (C‐2, C‐6), 116.79 (q, *J*=4.2 Hz, C‐6’). HRMS (ESI−): *m/z* [M−H]^−^ calculated for C_15_H_8_O_2_N_2_Cl_2_F_3_=374.9920, found: 374.9922.

#### 1‐(3,5‐Dichlorophenyl)‐3‐(3‐(Trifluoromethyl)phenyl)guanidine Hydrochloride (54)


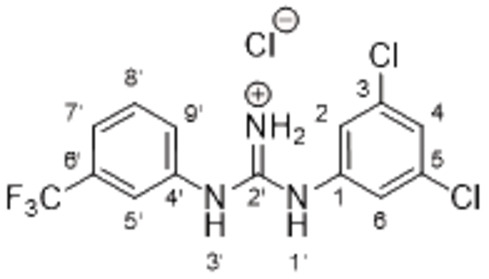


3‐(Trifluoromethyl)aniline (**45**, 400 mg, 2.5 mmol, 1.0 equiv) was dissolved in dry DCM (10 mL). Et_3_N (1.0 mL, 7.5 mmol, 3.0 equiv) and TCDI (442 mg, 2.5 mmol, 1.0 equiv) were added. After 2 h, 3,5‐dichloroaniline (402 mg, 2.5 mmol, 1.0 equiv) was added. The temperature was increased to 50 °C and the reaction mixture was stirred overnight. The solvent was removed under reduced pressure, the solid residue was dissolved in DMF, applied on flash chromatography column and separated (RP−C18aq, eluent water/methanol, gradient 0–100 %) to obtain the crude thiourea intermediate **63** (224 mg, 0.6 mmol). Solution of IBX (188 mg, 0.7 mmol, 1.1 eq) in aqueous ammonia (1.2 mL of 28–30 %) was added dropwise to the solution of the thiourea intermediate in acetonitrile (10 mL) and the mixture was stirred at RT for 1 h. The reaction mixture was evaporated, dissolved in DMF (2 mL) (with several drops of concentrated HCl added for full dissolution), applied on flash chromatography column and separated (RP−C18aq, eluent water/methanol, gradient 0–100 %) to obtain **54** (55 mg, 6 %) as a white solid. ^1^H NMR (DMSO, 401 MHz) δ 10.76 (2H, s, NH_2_), 8.45 (2H, s, H‐1’, H‐3’), 7.74–7.71 (1H, m, H‐5’), 7.67–7.59 (3H, m, H‐7’, H‐8’, H‐9’), 7.48 (1H, t, *J*=1.8 Hz, H‐4), 7.46 (2H, d, *J*=1.8 Hz, H‐2, H‐6). ^13^C NMR (DMSO, 101 MHz) δ 153.98 (C‐2’), 138.66 (C‐4’), 136.76 (C‐1), 134.45 (C‐3, C‐5), 130.70 (C‐8’), 130.08 (d, *J*=32.0 Hz, C‐6’), 128.01 (C‐9’), 125.41 (C‐4), 123.80 (d, *J*=272.8 Hz, CF_3_), 122.73 (d, *J*=3.9 Hz, C‐7’), 122.65 (C‐2, C‐6), 120.78 (d, *J*=3.9 Hz, C‐5’).

HRMS (ESI+): *m/z* [M+H]^+^ calculated for C_15_H_10_ON_4_Cl_2_F_3_=389.0178, found: 389.0181.

#### 5,6‐Dichloro‐6′‐(Trifluoromethyl)‐1 *H*,1′*H*‐2,2′‐Bibenzo[*d*]imidazole (55)


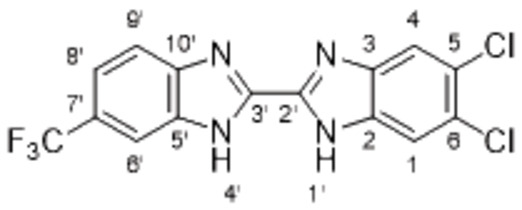


5‐(Trifluoromethyl)‐1*H*‐benzo[*d*]imidazole‐2‐carboxylic acid (**64**, 24 mg, 0.10 mmol, 1.0 equiv), 4,5‐dichlorobenzene‐1,2‐diamine (28 mg, 0.16 mmol, 1.5 equiv), HATU (52 mg, 0.14 mmol, 1.3 equiv), and Et_3_N (0.05 mL, 0.31 mmol, 3.0 equiv) were dissolved in dry DCM (3.0 mL) and the mixture was stirred at RT for 30 min. Volatiles were removed under reduced pressure, acetic acid (3.0 mL) was added to the solid residue and the mixture was heated overnight to 60 °C. Water (2 mL) was added and the mixture was applied on flash chromatography column and separated (RP−C18aq, eluent water/methanol, gradient 0–100 %) to obtain **55** (16 mg, 41 %) as a white solid. ^1^H NMR (DMSO, 500 MHz) δ 14.08 (2H, s, H‐1’, H‐4’), 8.01 (2H, s, H‐1, H‐4), 7.91 (1H, s, H‐8’), 7.84 (1H, s, H‐9’), 7.63 (1H, dd, *J*=8.6, 1.7 Hz, H‐6’). ^13^C NMR (DMSO, 126 MHz) δ 145.47 (C‐3’), 145.44 (C‐2’), 128.13 (C‐3), 125.96 (C‐6), 123.80 (C‐5), 121.64 (C‐2), 120.07 (C‐6’). HRMS (ESI−): *m/z* [M−H]^−^ calculated for C_15_H_8_N_4_Cl_2_F_3_=371.0073, found: 371.0071.

#### 5,6‐Dichloro‐*N*‐(3‐(Trifluoromethyl)phenyl)‐1*H*‐Benzo[*d*]imidazol‐2‐Amine (56)


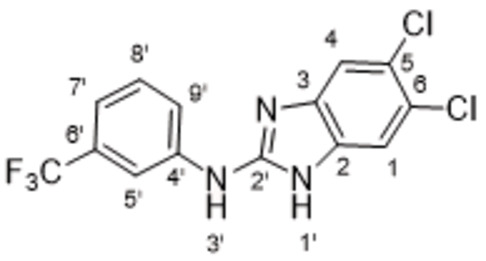


3‐(Trifluoromethyl)aniline (**45**, 100 mg, 0.62 mmol, 1.0 equiv) in dry DCM (5.0 mL) was added to the solution of TCDI (121 mg, 0.68 mmol, 1.1 equiv) and Et_3_N (0.26 mL, 1.9 mmol, 3.0 equiv) in DCM (5.0 mL) at 0 °C and stirred for 3 h (full conversion of starting compound into isothiocyanate was observed in UPLC‐MS). 4,5‐Dichlorobenzene‐1,2‐diamine (110 mg, 0.62 mmol, 1.0 equiv) was added to the reaction mixture and the mixture was heated to 60 °C for 24 h. Volatiles were removed under reduced pressure, the residue was dissolved in DMF, applied on flash chromatography column and separated (RP−C18aq, eluent water/methanol, gradient 0–100 %) to obtain **56** (51 mg, 24 %) as a yellowish solid. ^1^H NMR (DMSO, 401 MHz) δ 11.54 (1H, s, H‐3’), 8.03–7.99 (1H, m, H‐5’), 7.88 (1H, d, *J*=8.2 Hz, H‐9’), 7.67 (1H, dd, *J*=8.0, 8.0 Hz, H‐8’), 7.63 (2H, s, H‐1, H‐4), 7.53 (1H, d, *J*=7.8 Hz, H‐7’). ^13^C NMR (DMSO, 101 MHz) δ 149.83 (C‐2’), 138.33 (C‐4’), 132.52 (C‐2, C‐3), 130.70 (C‐8’), 130.18 (q, *J*=31.8 Hz, C‐6’), 124.87 (C‐9’), 124.65 (C‐5, C‐6), 123.97 (q, *J*=272.5 Hz, CF_3_), 120.96 (C‐7’), 117.57 (C‐5’), 113.60 (C‐1, C‐4). HRMS (ESI+): *m/z* [M+H]^+^ calculated for C_14_H_9_N_3_Cl_2_F_3_=346.0120, found: 346.0118.

#### 3,4‐Dichloro‐*N*‐(6‐(Trifluoromethyl)‐1*H*‐Benzo[*d*]imidazol‐2‐yl)benzamide (57)


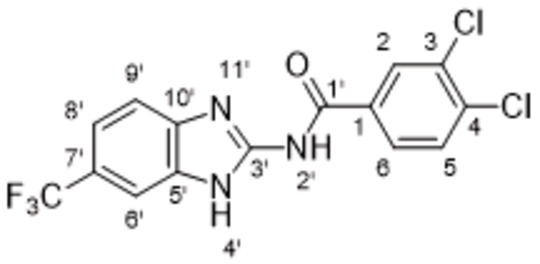


A mixture of 3,4‐dichlorobenzoic acid (**67**, 70 mg, 0.37 mmol, 1.0 equiv) and thionyl chloride (1.0 mL) was stirred for 1 h at 50 °C. Thionyl chloride was removed under reduced pressure, the residue was suspended in DCM (5.0 mL), 6‐(trifluoromethyl)‐1*H*‐benzo[*d*]imidazol‐2‐amine (74 mg, 0.37 mmol, 1.0 equiv) and Et_3_N (0.26 mL, 1.83 mmol, 5.0 equiv) were added, and the mixture was stirred for 1 h at RT. Volatiles were removed under reduced pressure, the residue was dissolved in DMF, applied on flash chromatography column and separated (RP−C18aq, eluent water/methanol, gradient 0–100 %) to obtain **57** (120 mg, 88 %) as a yellowish solid. ^1^H NMR (DMSO, 401 MHz) δ 8.39 (1H, d, *J*=2.1 Hz, H‐2), 8.11 (1H, dd, *J*=8.4, 2.1 Hz, H‐6), 7.90 (1H, d, *J*=1.7 Hz, H‐6’), 7.87–7.82 (1H, m, H‐5), 7.75 (1H, d, *J*=8.4 Hz, H‐9’), 7.59 (1H, dd, *J*=8.5, 1.8 Hz, H‐8’). ^13^C NMR (DMSO, 101 MHz) δ 165.75 (C‐1’), 148.69 (C‐3’), 135.71 (C‐4), 134.93 (C‐10’), 133.62 (C‐1), 132.20 (C‐5’), 131.56 (C‐3), 131.03 (C‐5), 130.46 (C‐2), 128.77 (C‐6), 124.76 (q, *J*=271.9 Hz, CF_3_), 123.44 (q, *J*=31.9 Hz, C‐7’), 119.85 (C‐8’), 114.29 (C‐9’), 110.89 (C‐6’). HRMS (ESI+): *m/z* [M+H]^+^ calculated for C_15_H_9_ON_3_Cl_2_F_3_=374.0069, found: 374.0070.

#### *N*‐(3,5‐Dichlorophenyl)‐6‐(Trifluoromethyl)‐1H‐Benzo[*d*]imidazole‐2‐Carboxamide (58)


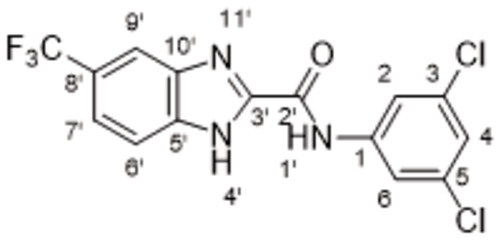


5‐(Trifluoromethyl)‐1*H*‐benzo[*d*]imidazole‐2‐carboxylic acid (**64**, 50 mg, 0.22 mmol, 1.0 equiv), 3,5–dichloroaniline (35 mg, 0.22 mmol, 1.0 equiv), HBTU (124 mg, 0.33 mmol, 1.5 equiv), and Et_3_N (0.09 mL, 0.65 mmol, 3.0 equiv) were dissolved in dry DMF (3.0 mL) and stirred overnight at RT. The reaction mixture was applied on flash chromatography column and separated (RP−C18aq, eluent water/methanol, gradient 0–100 %) to obtain **58** (16 mg, 20 %) as a white solid. ^1^H NMR (DMSO, 401 MHz) δ 11.44 (1H, s, H‐1’), 8.07 (2H, d, *J*=1.9 Hz, H‐2, H‐6), 8.04–8.02 (1H, m, H‐9’), 7.89 (1H, d, *J*=0.8 Hz, H‐6’), 7.66 (1H, dd, *J*=8.7, 1.8 Hz, H‐7’), 7.37 (1H, t, *J*=1.9 Hz, H‐4). ^13^C NMR (DMSO, 101 MHz) δ 157.36 (C‐2’), 147.31 (C‐3’), 140.55 (C‐1), 134.02 (C‐3, C‐5), 124.74 (q, *J*=271.7 Hz, CF_3_), 124.27 (q, *J*=31.5 Hz, C‐8’), 123.56 (C‐4), 120.47 (C‐7’), 118.75 (C‐2, C‐6), 116.98 (C‐6’), 114.65 (C‐9’). HRMS (ESI+): *m/z* [M+H]^+^ calculated for C_15_H_9_ON_3_Cl_2_F_3_=374.0069, found: 374.0066.

#### 1‐(5,6‐Dichloro‐1*H*‐Benzo[*d*]imidazol‐2‐yl)‐3‐(3‐(Trifluoromethyl)phenyl)urea (59)


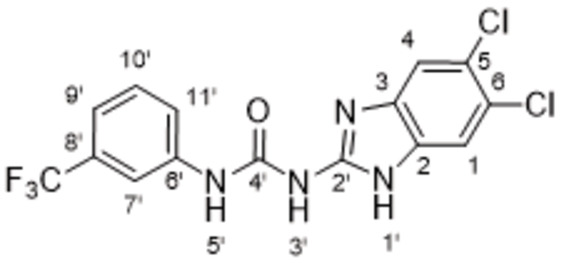


The treatment of compound **46** (~50 mg (179 mg of the crude mixture), 0.27 mmol, 1.0 equiv; prepared from **45)** and 5,6‐dichloro‐1*H*‐benzo[*d*]imidazol‐2‐amine (54 mg, 0.27 mmol, 1.0 equiv) according to Method B afforded **59** (21 mg, 20 %) as a yellowish solid. ^1^H NMR (DMSO, 401 MHz) δ 8.19 (1H, d, *J*=2.0 Hz, H‐7’), 7.69–7.64 (1H, m, H‐11’), 7.59–7.50 (3H, m, H‐1, H‐4, H‐10’), 7.39–7.34 (1H, m, H‐9’). ^13^C NMR (DMSO, 101 MHz) δ 141.23 (C‐2’), 140.18 (C‐5, C‐6), 134.21 (C‐6’), 129.95 (C‐1, C‐4), 129.56 (q, *J*=31.4 Hz, C‐8’), 124.21 (q, *J*=272.2 Hz, CF_3_), 122.33 (C‐11’), 118.68 (C‐9’), 114.78–114.58 (m, C‐7’), 114.03 (C‐10’). HRMS (ESI+): *m/z* [M+H]^+^ calculated for C_15_H_10_ON_4_Cl_2_F_3_=389.0178, found: 389.0178.

#### 1‐(3,5‐Dichlorophenyl)‐3‐(6‐(Trifluoromethyl)‐1*H*‐Benzo[*d*]imidazol‐2‐yl)urea (60)


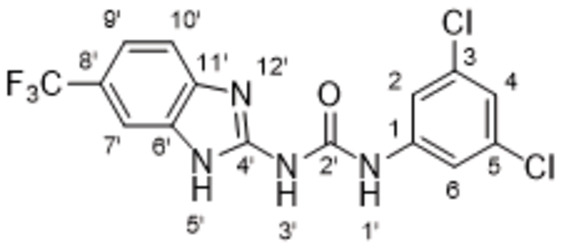


6‐(Trifluoromethyl)‐1*H*‐benzo[*d*]imidazol‐2‐amine (50 mg, 0.25 mmol, 1.0 equiv) was dissolved in dry THF (5.0 mL) and Et_3_N (0.10 mL, 0.75 mmol, 3.0 equiv) and Boc_2_O (54 mg, 0.25 mmol, 1.0 equiv) were successively added. The reaction mixture was stirred for 1 h at RT, solvent was removed under reduced pressure, and the residue was dissolved in dry DCM (5.0 mL). In parallel, 1,3‐dichloro‐5‐isocyanatobenzene (**69**) was prepared by the treatment of 3,5‐dichloroaniline (**68**, 40 mg, 0.25 mmol, 1.0 equiv) with Et_3_N (0.10 mL, 0.75 mmol, 3.0 equiv) and triphosgene (73 mg, 0.25 mmol, 1.0 equiv) in dry DCM (5.0 mL) at RT for 1 h. The two solutions were mixed and the reaction mixture was stirred at 50 °C overnight. Solvent was removed under reduced pressure and the residue was dissolved in TFA (5.0 mL) and stirred for 10 min at RT till full deprotection (UPLC‐MS). Volatiles were removed under reduced pressure till constant volume, the residue was dissolved in DMF, applied on flash chromatography column and separated (RP‐C18aq, eluent water/methanol, gradient 0–100 %) to obtain **60** (40 mg, 40 %) as a reddish solid. ^1^H NMR (DMSO, 401 MHz) δ 9.82 (1H, s, H‐1’), 7.71 (2H, d, *J*=1.9 Hz, H‐2, H‐6), 7.70–7.68 (1H, m, H‐7’), 7.56–7.52 (1H, m, H‐10’), 7.46–7.42 (1H, m, H‐9’), 7.19 (1H, t, *J*=1.9 Hz, H‐4). ^13^C NMR (DMSO, 101 MHz) δ 155.24 (C‐4’), 150.94 (C‐2’), 142.11 (C‐1), 134.08 (C‐3, C‐5), 133.57 (C‐11’), 129.03 (C‐6’), 124.99 (q, *J*=271.4 Hz, CF_3_), 122.01 (q, *J*=31.6 Hz, C‐8’), 121.28 (C‐4), 118.49 (q, *J*=3.8 Hz, C‐9’), 116.72 (C‐2, C‐6), 112.85 (C‐10’), 109.55 (C‐7’). HRMS (ESI+): *m/z* [M+H]^+^ calculated for C_15_H_10_ON_4_Cl_2_F_3_=389.0178, found: 389.0181.

#### Ethyl 2‐Oxo‐2‐((3‐(Trifluoromethyl)phenyl)amino)acetate (61)


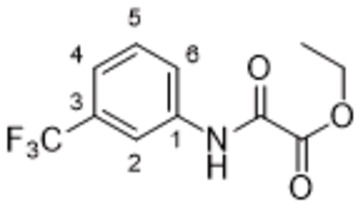


3‐(Trifluoromethyl)aniline (**45**, 0.5 g, 3.1 mmol, 1.0 equiv) and diethyl oxalate (1.4 g, 9.4 mmol, 3.0 equiv) were dissolved in toluene and heated to 90 °C for 2 days. Reaction mixture was concentrated till constant volume and the residues of diethyl oxalate were removed by high vacuum distillation to give **61** (800 mg, 99 %) as a yellowish solid. ^1^H NMR (DMSO, 401 MHz) δ 11.09 (1H, s, NH), 8.19 (1H, s, H‐2), 8.03 (1H, d, *J*=1.4 Hz, H‐6), 7.61 (1H, dd, *J*=8.0, 8.0 Hz, H‐5), 7.51 (1H, d, *J*=7.8 Hz, H‐4), 4.32 (2H, q, *J*=7.1 Hz, −C**H**_2_−CH_3_), 1.32 (3H, t, *J*=7.1 Hz, −CH_2_−C**H**_3_). ^13^C NMR (DMSO, 101 MHz) δ 160.17 (COO), 155.76 (CON), 138.34 (C‐1), 130.08 (C‐5), 129.45 (q, *J*=31.8 Hz, C‐3), 124.11 (C‐6), 123.98 (q, *J*=272.9 Hz, CF_3_), 121.09 (q, *J*=4.0 Hz, C‐4), 116.68 (q, *J*=4.1 Hz, C‐2), 62.61 (d, *J*=4.4 Hz, −**C**H_2_−CH_3_), 13.83 (−CH_2_‐**C**H_3_). MS (ESI+): *m/z* [M+H]^+^ calculated for C_11_H_11_O_3_NF_3_=262.1, found: 262.2.

#### 2‐Oxo‐2‐((3‐(Trifluoromethyl)phenyl)amino)acetic Acid (62)


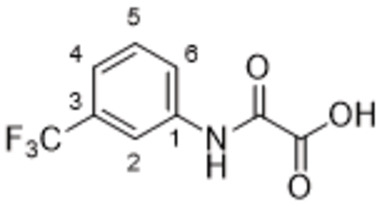


Compound **61** (300 mg, 1.15 mmol, 1.0 equiv) was dissolved in a mixture water/dioxane (0.5 mL/0.5 mL). The solution was cooled to 0 °C, LiOH.2H_2_O (145 mg, 3.45 mmol, 3.0 equiv) was added, and stirred for 2 h. The reaction mixture was quenched with aq. saturated NH_4_Cl solution. Solvent was removed under reduced pressure, the residue was dissolved in DMF, applied on flash chromatography column and separated (RP−C18aq, eluent water/methanol, gradient 0–100 %) to obtain **62** (254 mg, 94 %) as a yellowish solid. ^1^H NMR (DMSO, 401 MHz) δ 10.64 (1H, s, COO**H**), 8.30 (1H, s, H‐2), 8.03 (1H, dd, *J*=8.2, 2.1 Hz, H‐6), 7.53 (1H, dd, *J*=8.0, 8.0 Hz, H‐5), 7.39 (1H, d, *J*=7.8 Hz, H‐4). ^13^C NMR (DMSO, 101 MHz) δ 165.61 (COO), 162.06 (CON), 139.46 (C‐1), 129.77 (C‐5), 129.32 (q, *J*=31.5 Hz, C‐3), 124.18 (q, *J*=272.5 Hz, CF_3_), 123.24 (C‐6), 119.74 (q, *J*=4.4 Hz, C‐4), 115.60 (q, *J*=4.1 Hz, C‐2). MS (ESI−): *m/z* [M−H]^−^ calculated for C_9_H_5_O_3_NF_3_=232.0, found: 232.2.

### Protein‐Ligand Docking and Virtual Screening

Virtual screening was performed using Schrödinger Drug Discovery Suite version 2018–4 (Schrödinger, LLC, New York, NY, 2018). Structure of SARS‐CoV‐2 RdRp (PDB ID: 6 M71) was prepared for docking by Protein Preparation Wizard. It was protonated (neutral pH) and its energy was minimized. The library of 68,380 compounds from Maybridge Chemical Holdings Ltd was downloaded from ZINC database and their 3D structures were built using LigPrep. This was followed by their single precision docking using Glide. Finally, 100 compounds with the best score were inspected and 20 of them were selected based on visual inspection (to remove non‐drug‐like complexes) and scaffold diversity (Table S1).

### Biology

#### Protein Expression

Vectors for expression of 6×His‐TEV‐nsp7 (#154757), 6×His‐TEV‐nsp8 (#154758) and 6×HisMBP‐TEV‐nsp12 (#154759) were ordered from AddGene.^[22]^

6×His‐TEV‐Nsp7 and 6×His‐TEV‐Nsp8 were separately expressed in *E. coli* BL21 CodonPlus RIL. The cell culture was grown at 37 °C until reaching OD_600_ 0.6, after which the expression was induced by the addition of Isopropyl β‐D‐1‐thiogalactopyranoside to the final concentration of 0.4 mM. Bacterial cultures were then incubated for another 16 h at 18 °C. Afterward, the cells were harvested by centrifugation at 3,000×g for 15 min and resuspended in 50 mM Na‐HEPES, pH 7.4, 300 mM NaCl, 30 mM imidazole, 10 % (v/v) glycerol and 2 mM β‐mercaptoethanol with the addition of benzonase and protease inhibitor cocktail (Roche). The cells were then disrupted using a one‐shot homogenizer (Constant Systems) operating at 1.9 kbar. The obtained lysate was clarified by centrifugation at 50,000×g for 30 min and loaded onto HisTrap™ HP column (Cytiva) equilibrated in lysis buffer. The column was subsequently washed with a high‐salt buffer containing 1,000 mM NaCl and a low‐salt buffer with 150 mM NaCl. Finally, the his‐tagged proteins were eluted by 500 mM imidazole. To cleave off the purification tag, his‐tagged TEV protease was added in a 1 : 10 (w:w) ratio and the whole mixture was dialyzed against the low‐salt buffer for 16 h at 4 °C. Reverse IMAC was subsequently utilized to purify nsp7 or nsp8 of uncleaved constructs, TEV protease and cleaved purification tags. The flowthrough fractions were dialyzed against storage buffer (20 mM Na‐HEPES pH 7.4, 150 mM NaCl, 5 % (v/v) glycerol, 1 mM TCEP). The proteins were finally concentrated to 484 μM for nsp7 and 221 μM for nsp8 using an Amicon® Ultra Centrifugal Filter, 3 kDa MWCO (Millipore) and stored at −80 °C.

*Sf9* insect cell line was employed to produce nsp12. Bac‐to‐Bac™ system (Invitrogen) was utilized to generate the 6×His‐MBP‐TEV‐nsp12 bacmid and the insect cells were then transfected and the supernatant containing the recombinant baculoviruses was harvested after 5 days. The expression was initiated by infecting the cell suspension (3×10^6^ cells/ml) with the stock solution of baculoviruses in a 1 : 100 ratio. Subsequently, the culture was incubated at 27 °C for 96 h. After expression, the cells were harvested by centrifugation at 3,000×g for 15 min, resuspended in a lysis buffer containing 50 mM Na‐HEPES pH 7.4, 300 mM NaCl, 30 mM imidazole, 3 mM MgCl_2_ 10 % (v/v) glycerol, 5 mM β‐mercaptoethanol and disrupted by one‐shot homogenizer (Constant Systems). Before disruption benzonase and protease inhibitor cocktail (Roche) were added. The lysate was subsequently cleared by centrifugation at 70,000×g for 30 min and ultracentrifugation at 235,000×g for 60 min and applied onto an HisTrap™ HP column (Cytiva) in lysis buffer. The column was washed with buffer containing 1,000 mM NaCl. An MBPTrap™ column (Cytiva) was then connected downstream to the HisTrap™ column and both columns were washed with 500 mM imidazole to elute His‐tagged proteins onto the MBPTrap™. The HisTrap™ column was subsequently disconnected and the MBPTrap™ was washed with additional 5 column volumes of lysis buffer. Upon elution with 116.9 mM maltose, 6×His tagged TEV protease was added in a 1 : 10 (w:w) ratio and the sample was dialyzed against 3 liters of lysis buffer for 16 h at 4 °C. Uncleaved protein, 6×His‐MBP tag and protease were subsequently removed on an HisTrap™ column and the flowthrough fraction containing nsp12 was diluted to 80 mM NaCl using heparin dilution buffer (20 mM Na‐HEPES pH 7.4, 1 mM MgCl_2_, 10 % (v/v) glycerol, 5 mM β‐mercaptoethanol. The sample was then loaded onto HiTrap Heparin HP (Cytiva) equilibrated in heparin dilution buffer containing 80 mM NaCl. Nsp12 was eluted with a linear gradient from 80 mM to 1,000 mM NaCl. Peak fractions were pooled and dialyzed against 3 liters of storage buffer (20 mM Na‐HEPES pH 7.4, 300 mM NaCl, 1 mM MgCl_2_, 10 % (v/v) glycerol, 1 mM TCEP). Finally, the sample was concentrated in an Amicon® Ultra Centrifugal Filter, 50 kDa MWCO (Millipore) to the final concentration of 21.5 μM and stored at−80 °C as single‐use aliquots.

#### The Fluorescence‐Based RdRp Polymerase Assay

The activity of the RNA‐dependent RNA polymerase complex in real time was monitored using a SYBR Green I‐based approach. First, the reaction mixture was prepared containing reaction buffer (20 mM HEPES pH 8, 10 mM KCl, 6 mM MgCl_2_, 0.01 % Triton X‐100, 1 mM DTT), 250 μM ATP, 5 μM SYBR Green I and RdRp complex composed of nsp12, nsp7 and nsp8 at the ratio of 1 : 3 : 3 (nsp12:nsp7:nsp8) to the final concentration of 0.63 μM:1.88 μM:1.88 μM. The reaction mixture was aliquoted in 17 μl into the 96‐well plate with the final reaction volume being 20 μl. In the case of the inhibitor testing, the compound or solvent, respectively, was added into 17 μl providing the final concentration of DMSO at 5 %. The reaction was initiated by adding a dsRNA template which was prepared by annealing of ssRNA template (5'‐_58_UAUAACUUAAUCUCACAUAGC‐3') and ssRNA primer (5'‐GCUAUGUGAGAUUAAGUUAU‐3'). All components of the annealing reaction, namely annealing buffer (10 mM Na‐HEPES pH 8.0, 25 mM NaCl, 2.5 mM EDTA), ssRNA template and ssRNA primer were mixed, and heated up for 5 min at 94 °C. The mixture was then slowly cooled down to room temperature and stored at −80 °C. After adding the dsRNA template into the reaction to the final concentration of 0.5 μM, the 96‐well plate was transferred into the thermocycler QuantStudio 5 real‐time PCR system (Applied Biosystems) and the reaction was incubated for 50 min at 30 °C. The fluorescence of SYBR Green I enhanced after binding on the nascent dsRNA molecule was recorded each 1 min and plotted against the time. After background subtraction, the area under the curve (AUC) for every reaction was estimated using a midpoint method for numerical integration. The residual RdRp activity in % was obtained as AUC_i_/AUC_n_×100, where AUC_i_ and AUC_n_ represent AUC for reactions with and without an inhibitor, respectively.

#### The Gel‐Based RdRp Polymerase Activity Verification

The FAM label RNA primer (5' FAM‐GCUAUGUGAGAUUAAGUUAU‐3') and the RNA template were mixed in annealing buffer and heated at 94 °C for 5 min after which the mixture was cooled down to room temperature. The RdRp polymerase mixtures were subsequently prepared identically as for the fluorescence‐based assay with the only difference being the RdRp protein concentrations set at 5 μM nsp12, 15 μM nsp7 and 15 μM nsp8. Reaction mixtures were then incubated for 50 min at 30 °C. Subsequently, the reactions were stopped by adding TBE buffer (90 mM Tris‐base pH 8.0, 90 mM boric acid, 2 mM EDTA) with 50 mM EDTA, 7  M urea and proteinase K, each mixture was heated to 95 °C for 5 min and loaded on 20 % Acrylamide gel with 7 M urea in TBE buffer.

#### Antiviral and Cytotoxicity Determination

The anti‐SARS‐CoV‐2 activity was measured by determining the extent to which the test compounds inhibited virus‐induced cytopathic effect in Calu‐3 cells. Briefly, two‐fold fold serial dilutions of compounds were added in triplicate in a 384‐well plate with 15,000 Calu‐3 cells in DMEM medium with 2 % FBS, 100 U of penicillin/ml and 100 μg of streptomycin/ml (all Merck). After 1 h incubation SARS‐CoV‐2 was added at multiplicity of infection 0.01. Following three days incubation at 37 °C in 5 % CO_2_ the cell viability was determined by addition of XTT solution (Sigma‐Aldrich) for 4 h and the absorbance was measured using EnVision plate reader (Perkin Elmer). Drug concentrations required to reduce viral cytopathic effect by 50 % (EC_50_) were calculated using nonlinear regression from plots of percentage cell viability versus log_10_ drug concentration using GraphPad Prism v.9.0.0 (GraphPad Software). Cytotoxicity was evaluated by incubating the same two‐fold serial dilutions of each compound with Calu‐3 cells as above but without virus. Following three days of incubation at 37 °C in 5 % CO_2_, the cell viability was determined by the addition of XTT solution as above. The compound concentrations resulting in a 50 % reduction of absorbance (CC_50_) were calculated from plots of the percentage of absorbance versus log_10_ drug concentration as above.

## Conflict of Interests

The authors declare no conflict of interest.

1

## Supporting information

As a service to our authors and readers, this journal provides supporting information supplied by the authors. Such materials are peer reviewed and may be re‐organized for online delivery, but are not copy‐edited or typeset. Technical support issues arising from supporting information (other than missing files) should be addressed to the authors.

Supporting Information

## Data Availability

The data that support the findings of this study are available from the corresponding author upon reasonable request.
