# Supplementary material for: Developing Allosteric Inhibitors of SARS‐CoV‐2 RNA‐Dependent RNA Polymerase
Source: ChemMedChem. 2024 Oct 3;19(23):e202400367. doi: 10.1002/cmdc.202400367 (PMC11617668; doi:10.1002/cmdc.202400367)
Supplement: Supplementary file 1 — Supporting Information [file CMDC-19-e202400367-s001.pdf]

ChemMedChem

Supporting Information

Developing Allosteric Inhibitors of SARS-CoV-2 RNA-Dependent RNA Polymerase

Artem Chayka, Matěj Danda, Alžběta Dostálková, Vojtěch Spiwok, Anna Klimešová, Marina Kapisheva, Michala Zgarbová, Jan Weber, Tomáš Ruml, Michaela Rumlová,* and Zlatko Janeba*

SUPPORTING INFORMATION

Developing Allosteric Inhibitors of SARS-CoV-2 RNA-dependent RNA polymerase

Artem Chayka,[a] Matěj Danda,[b] Alžběta Dostálková,[b] Vojtěch Spiwok,[c] Anna Klimešová,[b] Marina Kapisheva,[b] Michala Zgarbová,[a,d] Jan Weber,[a] Tomáš Ruml,[c] Michaela Rumlová,*[b] and Zlatko Janeba*[a]

[a] *Institute of Organic Chemistry and Biochemistry of the Czech Academy of Sciences, Flemingovo nám. 2, 160 00 Prague 6, Czech Republic*

[b] *Department of Biotechnology, University of Chemistry and Technology, Prague, Technická 5, 166 28 Prague 6, Czech Republic*

[c] *Department of Biochemistry and Microbiology, University of Chemistry and Technology, Prague, Technická 5, 166 28 Prague 6, Czech Republic*

[d] *Department of Genetics and Microbiology, Charles University, Faculty of Sciences, Viničná 5, 128 44 Prague 2, Czech Republic*

**michaela.rumlova@vscht.cz; *janeba@uochb.cas.cz*

TABLE OF CONTENTS

Table S1. Structures and scoring of 20 compounds (compounds **1** and **A**–**S**) selected from the virtual screening of 68,380 commercially available compounds. ……..…………………………..… S2

Table S1. Structures and scoring of 20 compounds (compounds **1** and **A–S**) selected from the virtual screening of 68,380 commercially available compounds.

Compound	Structure	S (docking score)
**1**	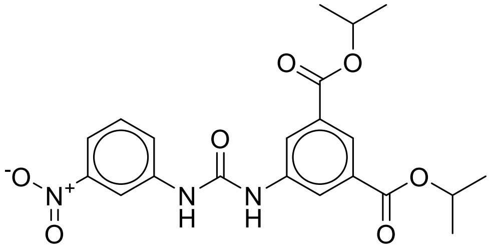	-7.8295808
**A**	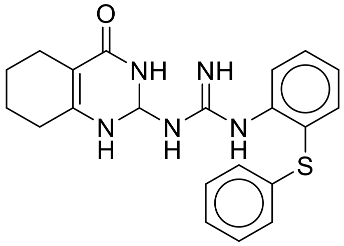	-7.0703621
**B**	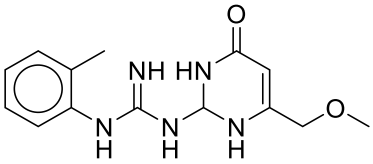	-7.0434899
**C**	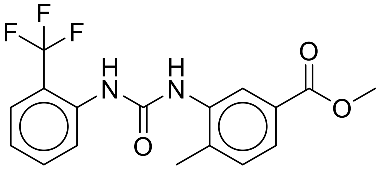	-6.567472
**D**	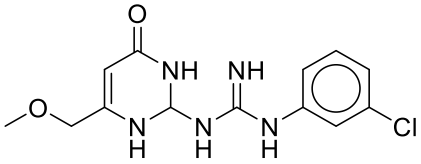	-6.4771843
**E**	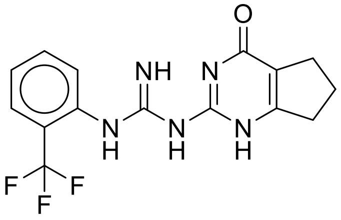	-6.4524002
**F**	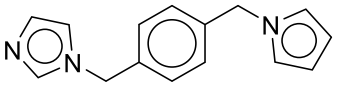	-6.261766
**G**	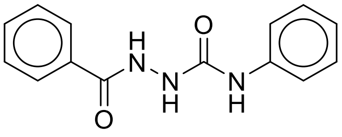	-6.2322345

**H**	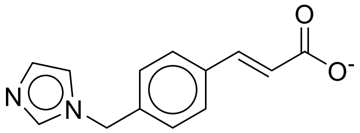	-6.1028147
**I**	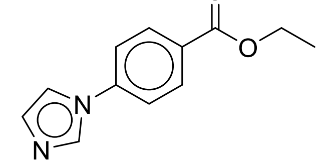	-5.8007474
**J**	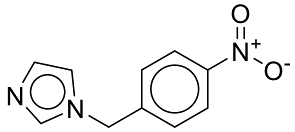	-5.7623787
**K**	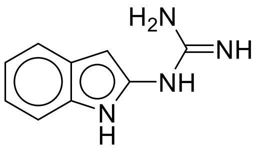	-5.5397496
**L**	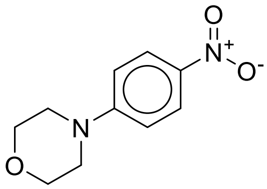	-5.5175824
**M**	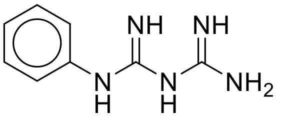	-5.4822135
**N**	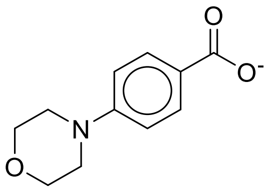	-5.4163342
**O**	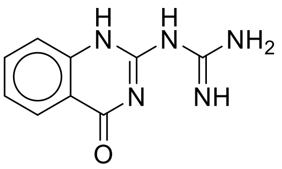	-5.3747888
**P**	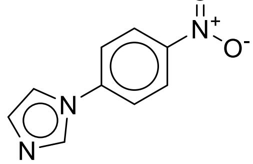	-5.2311282
**Q**	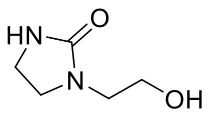	-5.063302

**R**	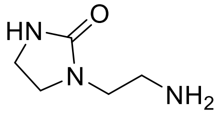	-5.0286021
**S**	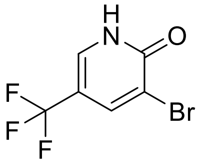	-4.9434032
